# Epidemiology of a SKin Ulceration Disease (SKUD) in the sea cucumber *Holothuria scabra* with a review on the SKUDs in Holothuroidea (Echinodermata)

**DOI:** 10.1038/s41598-020-78876-0

**Published:** 2020-12-17

**Authors:** Jérôme Delroisse, Kévin Van Wayneberghe, Patrick Flammang, David Gillan, Pascal Gerbaux, Noel Opina, Gildas Georges Boleslas Todinanahary, Igor Eeckhaut

**Affiliations:** 1 Biology of Marine Organisms and Biomimetics, Research Institute for Biosciences, University of Mons – UMONS, Place du Parc, 6, 7000 Mons, Belgium; 2 Marine Station of Belaza, Institut Halieutique et des Sciences Marines (IH.SM), University of Toliara, Route du Port Mahavatse II, P.O. Box 141, 601 Toliara, Madagascar; 3 Proteomics and Microbiology Lab, Research Institute for Biosciences, University of Mons – UMONS, Place du Parc, 6, 7000 Mons, Belgium; 4 Organic Synthesis and Mass Spectrometry Lab, Interdisciplinary Center for Mass Spectrometry, Research Institute for Biosciences, University of Mons – UMONS, Place du Parc, 6, 7000 Mons, Belgium; 5 Madagascar Holothurie (R&D of Indian Ocean Trepang), Toliara, Route du Port Mahavatse II, P.O. Box 141, 601 Toliara, Madagascar

**Keywords:** Zoology, Marine biology

## Abstract

Aquacultivated sea cucumbers often suffer from SKin Ulceration Diseases (SKUDs). SKUDs have been observed in six holothuroid species from nine countries. All SKUDs present a similar symptom—the skin ulceration—and can be induced by bacteria, viruses, or abiotic factors. We here provide an update on SKUDs in holothuroids and analyse the case of the SKUD observed in *Holothuria scabra* in Madagascar. Field observations revealed a seasonality of the disease (i.e. wintertime maximum peak). Morphological analyses of integument ulcers showed that sea cucumbers react by forming a collagen fibre plug. Metagenomic analyses revealed a higher proportion of Vibrionaceae (Gammaproteobacteria) in ulcers in comparison to the healthy integument of the same individuals. Experimental infection assays were performed with ulcer crude extracts and bacteria isolated from these extracts (e.g. *Vibrio parahaemolyticus*) but did not significantly induce skin ulceration. Our results suggest that the disease is not induced by a pathogen or, at the very least, that the pathogen is not found within the ulcers as the disease is not transmissible by contact. An initial cause of the SKUD in Madagascar might be the repeated and prolonged exposures to cold temperatures. Opportunistic bacteria could settle in the dermis of ulcerated individuals and promote the ulcer extension. We propose a general nomenclature for SKUDs based on the acronym of the disease, the affected sea cucumber species (e.g. Hs for *Holothuria scabra*), the concerned region using an ISO code 3166-2 (e.g. MG for Madagascar), the description date (e.g. 20 for the year 2020), and, when known, the inducing agent (first letter of the general taxon, *b* for bacteria, *v* for virus in currently known cases; a *a* if it is an abiotic inducing parameter; nothing if the inducing cause has not been precisely identified). The disease described in this work will be designated under the name SKUD Hs-MG-20.

## Introduction

SKin Ulceration Diseases are observed in various aquacultivated holothuroids in different parts of the world. The diseases, characterised by skin ulcerations (i.e. alteration of the body wall), are grouped under the term SKUDs or Skin Ulceration Syndromes (SUS). The first report of a SKUD appeared in the work of Zhang and Liu^1^ who reported that *Apostichopus japonicus* juveniles were susceptible to this disease, which tends to occur as a result of high temperatures and high stocking densities. The SKUD spread rapidly from diseased individuals to healthy ones, making it difficult to control. If SKUD-affected animals are not eliminated from the broodstock, up to 95% of the reared individuals died^2^. Occasionally, an entire population was wiped out in a short time, once the disease sets in. Two years after the report of Zhang and Liu^1^, Morgan published a short note on a similar disease affecting adults of a tropical species, *Holothuria scabra, *on Bribie Island (Australia)^2^. The SKUD is associated to a loss of epidermal pigmentation within the epidermis as well as a abundant mucus secretion. The ulcers initially appeared around the mouth and/or the cloacum and spread on oral and ventral sides of the individuals before possibly reaching the whole integument surface. Becker et al*.*^3^ described the histopathology of a SKUD affecting *H. scabra* juveniles in a hatchery in Madagascar. The authors observed that the SKUD always begins by the appearance of a white lesion near the cloacal orifice^3^. The initial ulcer then extends onto the whole surface of the integument^3^. It destroys the epidermis, the connective tissue and also affect the ossicles^3^. The disease may quickly expand in the reared populations. A SKUD has also been recently described in *Holothuria arguinensis*, a potential European candidate for aquaculture^4^. Besides these three species, many other species studied as part of aquaculture development (e.g. *Isostichopus badionotus* in Colombia, *Holothuria forskali* in France) are prone to skin ulceration (Eeckhaut *pers. obs.*).

The cause of SKUD may be biotic (i.e. due to a pathogen) or abiotic (i.e. due to environmental changes and without the involvement of a pathogen). Concerning biotic causes, two types of agents are mentioned in the literature: a viral agent^5,6^ or several bacterial species, with some researches on *Apostichopus japonicus* showing that both can induce skin ulceration in this species. A bacterial cause has sometimes been suggested without demonstrating the causal effect of the bacterial infection in *A. japonicus*^1,7^*, H. arguinensis*^4^ and *H. scabra*^2,8–10^. In many cases, bacteria have only been identified in ulcers, but only few infection experiment assays have been performed (Table 1). Generally, bacterial identification is made on a culture basis accompanied by 16S rDNA gene sequencing of the cultured bacteria. In Becker et al*.*’s research^3^, several bacteria have also been identified in the skin ulcers of *H. scabra* juveniles but none of the multiple experimental infection tests from the isolated bacteria allowed to induce the SKUD suggesting to the authors that the identified bacteria were probably opportunistic and were taking advantage of the ulcers to proliferate. The identification of SKUD-inducing bacteria, after an experimental infection, has only been demonstrated in *A. japonicus*^5,6,11–14^. In this species, the development of a SKUD is carried out by immersing healthy individuals in water contaminated by an isolated bacterium or by injecting a solution enriched with the bacteria into the coelom or in the body wall. A dozen species of bacteria are present in ulcerations (Table 1) with *Vibrio spp* (Proteobacteria, Gammaproteobacteria) being the most commonly observed genus in lesions in *A. japonicus*^2,5,6,13–18^ and *H. scabra*^9,10^ (e.g. *Vibrio alginolyticus, Vibrio splendidus*). Besides bacteria, viruses have also been isolated from *A. japonicus* ulcers and can also induce SKUD if they are injected into the coelom or if healthy individuals are introduced into water contaminated with these viruses^5,6^.

**Table 1:** SKin Ulceration Diseases in sea cucumbers, the associated pathogens and the potential causes. Legend: Species; *H. sca.*: *Holothuria scabra; A. jap.: Apostichopus japonicus; H. arg.: Holothuria arguinensis; Pseudoalteromonas: P.; Vibrio: V.;* Stages; J: juveniles; A: adults; Ref.: references*.*

Pathogen(s)/cause(s)	Species	Stage(s)	Experimental induction of SKUD		Ref
			Assay(s)	Result(s)	
**Studies investigating the impact of biotic factors (bacteria, virus…)**					
*Arcobacter bivalviorum, P. citrea, P. sp., V. azureus, V. fortis, V. owensii, V. parahaemolyticus, V. rotiferianus, V. tubiashi, V. sp.*	*H. sca*	J	No assay (Detection only)		^8^
*V. alginolyticus*	*H. sca*	J	No assay (Detection only)		^9^
*V. salmonicida*	*H. sca*	A	No assay (Detection only)		^10^
*V. harveyi*	*H. sca*	A	No assay (Detection only)		^2^
*Bacterium* from the CFB group, *Roseobacter sp.*, *V. sp*., *V. natriegens*, *V. harveyi, V. alginolyticus*	*H. sca*	J	(*i*) Immersion (*ii*) Contact	No induction	^3^
*V. gigantis, V. crassostreae, P. spongiae, Alteromonas mediterranea*	*H. arg*	A	No assay (Detection only)		^4^
Unidentified Bacteria	*A. jap*	A	Immersion^2^	SKUD induction	^5^
Bacteria from CFB group, Alteromonadales, Vibrionales	*A. jap*	A	No assay (Detection only)		^7^
*V. cyclitrophicus, V. splendidus, V. harveyi, V. tasmaniensis, Photobacterium sp., Arthrobacter protophormiae, Staphylococcus equorum*	*A. jap*	A	(*i*) Body wall injection^1^ (*ii*) Immersion	SKUD induction	^12^
*Shewanella marisflavi*	*A. jap*	A	(*i*) Body wall injection (*ii*) Intracoelomic injection	SKUD induction	^18^
*P. sp., P. tetraodonis*	*A. jap*	A	(*i*) Immersion (*ii*) Intracoelomic injection	SKUD induction	^6^
*V. splendidus, Shewanella sp., P. tetraodonis*	*A. jap*	A	Intracoelomic injection	SKUD induction	^11^
*V. splendidus*	*A. jap*	A	Immersion	SKUD induction	^13,14^
Unidentified virus	*A. jap*	A	Immersion^2^	SKUD induction	^5^
Unidentified virus	*A. jap*	A	(*i*) Immersion (*ii*) Intracoelomic injection	SKUD induction	^6^
**Studies investigating the impact of abiotic factors (food quality, salinity, temperature…)**					
High temperatures, High stocking densities	*A. jap*	A	No assay		^1^
Non-adequate food	*H. sca*	A	Ingestion	SKUD induction	^17^
Low salinity (i.e. 20‰)	*H. sca*	A	Immersion	SKUD induction	^19^

SKUDs can also be induced without pathogens. Temperature and salinity were suggested to have a potential impact on the induction of SKUD^1,19^. In addition, autoclaved sediments enriched with different types of organic matter can induce skin ulceration in *H. scabra* when eaten by the sea cucumber^17^. The various tested organic matters added to the sediment in Eeckhaut et al*.*’s experiments were: (*i*) crushed integument ulcers coming from ulcerated *H. scabra*, (*ii*) crushed integument coming from healthy *H. scabra* juveniles, (*iii*) crushed skin from healthy fishes and (*iv*) crushed healthy red seaweed^17^. The three first types of organic matters induce skin ulceration in less than three days of feeding, sometimes leading to the death of individuals. The last, non-animal, organic matter did not induce skin ulcers.

**Figure 1:**
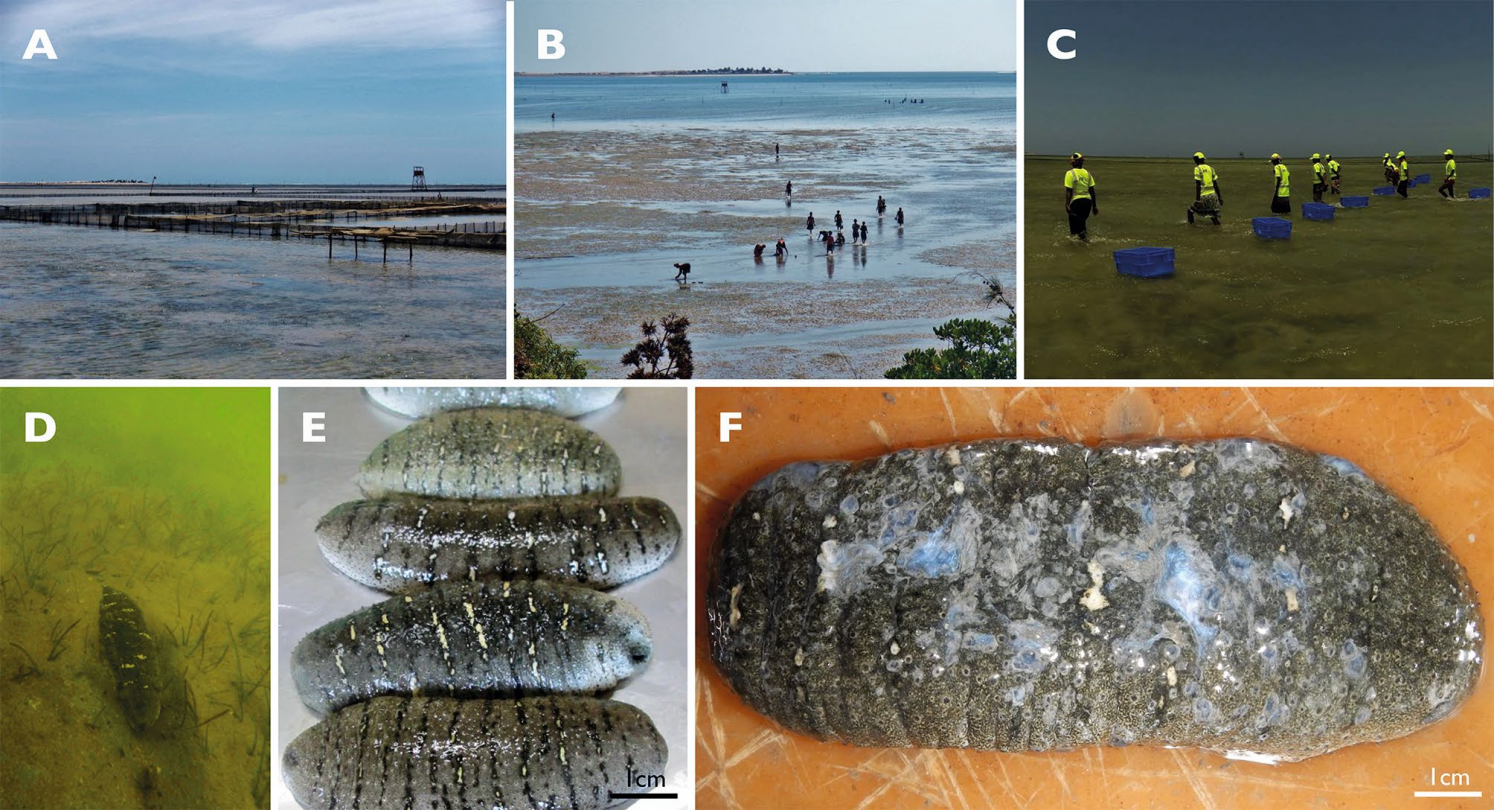
Holothuriculture in Toliara. (**A**) Pens from the company Indian Ocean Trepang at « La Mangrove » during low tide, (**B**) Collectors at « La Mangrove », (**C**) SKUD-affected collection at « La Mangrove », (**D**) *H. scabra* buried in the sediment, (**E**) Freshly collected sea cucumbers, (**F**) SKUD-affected sea cucumbers.

In Madagascar, in addition to the skin ulceration described by Becker et al.^3^ on juveniles reared in a hatchery, a SKin Ulceration Disease is also affecting adults of *H. scabra* grown in sea pens from the company Indian Ocean Trepang since 2016 (Fig. 1). This disease begins with skin ulcerations and causes the death of many individuals. The present research analyses the epidemiology of this disease through (*i*) a census of individuals affected over two years, (*ii*) a fine morphological approach of the skin ulceration symptoms, (*iii*) a characterisation of the microbiota taxa via shotgun metagenomic analyses and (*iv*) an in vivo approach where experimental infection assays were performed with crude extracts from sea-cucumber ulcerations and with bacteria isolated from these extracts.

## Material and methods

### Descriptive epidemiology

Sea cucumber aquaculture in Madagascar is only developed by one private company, Indian Ocean Trepang (IOT). The company is based in Toliara and produces millions of juveniles in a hatchery that are grown in sea pens with the help of villagers-farmers. *H. scabra* is the only aquacultivated species in this context. The process involves four main stages: (*i*) oocytes fertilisation in the laboratory, (*ii*) larval rearing in a hatchery, (*iii*) pre-growing in land-based ponds and (*iv*) growing in sea pens. Juveniles enter the sea at the size of 6 cm (about 15 g) and stay there for 8 to 14 months, until they reach a marketable size. They are then transformed into trepang for export to Hong Kong. IOT has various areas at sea where there are enclosures, the main one being near the village of Sarodrano where there are 127 hectares of enclosures.

A SKUD disease has been declared since 2016 in the IOT pens of Sarodrano and sick individuals were collected for elimination allowing a census of the number of affected individuals per month. Counts of diseased individuals were carried out at night at low tides at the full and new moon by a team of 10 people. Sea cucumbers were checked and their health status was directly verified on site. SKUD-affected individuals were then counted and removed from the pens. Data were collected from April 2016 to October 2017 to evaluate potential trends related to the period of the year. In parallel, temperatures were recorded in the IOT pens on a monthly basis.

### Analytical epidemiology

#### *Holothuria scabra *sea cucumber samplings

Individuals of *Holothuria scabra* Jaeger, 1833 were collected in the sea pens of Indian Ocean Trepang in 2017. For histology and scanning electron microscopy, sea cucumbers were dissected directly on site and tissues of interest were fixed in Bouin’s fluid or in a 3% solution of glutaraldehyde in cacodylate buffer (as described in^20^), respectively. For metagenomic analyses, samples were fixed in absolute ethanol (i.e. 100%). Samples were then transported to the Belgian laboratory (Biology of Marine Organisms and Biomimetics, UMONS) for further analyses. For in vivo experiments, sea cucumbers were transported to the aquaria of the “Institut Halieutique et des Sciences Marines” (IHSM) in Toliara.

#### Morphological analyses of the Skin Ulcerations of *Holothuria scabra*

In a healthy state, the body wall of *H. scabra* sea cucumbers consists of a monostratified epidermis covered by a cuticle, a sub-epidermal connective tissue layer, a layer of circular musculature and a mesothelium^21^. Two nerve plexi, one at the base of the epidermis and the other at the base of the mesothelium, are also present^22^. To better understand what is happening in the ulcerated tissues, we compared the upper body wall (epidermis and subepidermal connective tissue) of healthy individuals with ulcers of SKUD-affected individuals (stage II, see Results “Symptoms of SKUD-affected Holothuria scabra sea cucumbers in the southwest of Madagascar” section for the description of the stages) using light and electron microscopies.

After fixation for 12 h in Bouin’s fluid, tissues were decalcified during up to 5 days in a 1:1 mixture of a 2% ascorbic acid solution and NaCl 0.3 M at 4 °C^23^, dehydrated through graded series of ethanol and embedded in paraffin using a routine method^20,24^. Paraffin sections, 5–7 µm in thickness, were cut using a Microm HM340E microtome, mounted on clean glass slides and stained with Masson’s trichrome stain^24^.

For transmission electron microscopy (TEM), dorsal integument pieces were fixed for 3 to 24 h in 3% v/v glutaraldehyde in cacodylate buffer (0.1 M, pH 7.8; adjusted to 1,030 mOsm L^-1^ with NaCl) at 4 °C, rinsed in the same buffer, and post-fixed for 30 min in 1% w/v osmium tetroxide in the same buffer. After a final buffer wash, the pieces were decalcified for 5 days at 4 °C in a 1:1 mixture of 2% ascorbic acid and 0.3 M NaCl^23^. Decalcified pieces were dehydrated in graded ethanol and embedded in Spurr resin. Ultrathin sections (i.e. 80–90 nm) were cut with a Leica UCT ultramicrotome equipped with a diamond knife, collected on copper grids, and contrasted with uranyl acetate and lead citrate^25^ before observation with a Zeiss LEO 906E transmission electron microscope.

For scanning electron microscopy (SEM), pieces of the dorsal integument of *H. scabra* were fixed and dehydrated as described for classical histology. They were then dried using the critical-point method (using CO_2_ as a transition fluid), coated with gold in a sputter coater and observed with a JEOL JSM-6100 scanning electron microscope.

#### Shotgun metagenomic analyses of SKUD-affected dorsal integument of *Holothuria scabra*

##### DNA extractions

The total community DNA was extracted using the Blood and Tissue DNA Isolation Kit (QIAGEN, kit designed for the extraction of total DNA from animal blood and tissues and from cells, yeast, bacteria, or viruses) according to the manufacturer’s instructions. The purity of the extracted DNA was checked using a Denovix DS-11 UV–Vis microspectrophotometre (DeNovix Inc., Wilmington, DE, USA). The integrity of the DNA was confirmed by electrophoresis on a 1% agarose gel. All DNA samples were stored at − 20 °C until used in downstream analyses. Two sick individuals were investigated. One ulcerated area and one non-ulcerated area were selected for both individuals and genomic DNA was extracted for these 4 samples (i.e. ulcerated integument individual 1/replicate 1, ulcerated integument individual 2/replicate 2, healthy integument individual 1/replicate 1, healthy integument individual 2/replicate 2).

##### Metagenomic DNA sequencing and bioinformatic analysis

Shotgun metagenome libraries were prepared and sequenced on Illumina NovaSeq 6000 platform (2 × 150 bp) at Eurofins Genomics (www.eurofinsgenomics.eu). Raw data were uploaded as FASTQ after demultiplexing of paired-end reads. Reads generated after quality processing (i.e. dynamic trimming: minimum quality = 15, maximum low-quality base pairs = 5; detection and removal of adapter sequences using Skewer with default parameters^26^) and deduplication by MG-RAST 4.0.3 (Rapid Annotation using Subsystems Technology for Metagenomes) pipeline analysis^27–29^ were subjected to taxonomic analyses.

Taxonomic assignments and the overall microbial diversity within the samples were assessed by querying the reads of metagenomes against the Refseq database^30^ using the MG-RAST analysis tool using specific parameters (alignment length > 15 bp, e-value: e^−10^ to e^−40^, identity > 70%). Results from the query of all reads against the Refseq database were then filtered based on various taxonomic ranks (e.g. domain, phylum, class, …).

### In vivo experimental epidemiology

The in vivo experiments focused on the evaluation of the dorsal integument potential ulceration or recovery (i.e. wound healing of the integument surface) of *H. scabra* individuals placed in the context of a challenge by living bacteria isolated from skin ulcers of diseased sea cucumbers or by contact with a solution made of crushed SKUD-affected sea cucumbers.

#### Isolation of bacteria present in the skin ulcerations of *Holothuria scabra* and molecular identification of bacterium taxa

SKUD-affected integument ulcer fragments were soaked in liquid medium for cultivation of heterotrophic marine bacteria (Marine Broth 2216, Difco) during 12 h at 27 °C. The medium was mainly composed of peptone (5 g L^−1^), yeast extract (1 g L^−1^) and NaCl (19.45 g L^−1^) and was made with MilliQ water. Liquid cultures were then used to inoculate culture plates made using the same medium enriched with agar (i.e. Marine Agar 2216, Difco, 15 g L^−1^ of agar). After 24 h of incubation at 27 °C, colonies were isolated and streaked two more times on new agar plates to ensure purity. Three purified cultures were prepared (bacterial cultures 1, 2 and 3).

Isolated bacteria were used for body wall inoculations, intra-coelomic injections and oral force-feeding (“Attempted inductions of the SKin Ulceration Disease in healthy *Holothuria scabra* sea cucumbers” section) and were also preserved in absolute ethanol for identification. Bacterial identification was performed by Sanger DNA sequencing. Bacterial DNA from 5 to 10 mg subsamples was extracted using the Gram-negative adapted protocol of the DNeasy Blood & Tissue Kit (Qiagen). A 16S rRNA gene fragment of around 400 bp (V3–V4 hypervariable regions) was amplified by PCR using the PuReTaq Ready-To-Go PCR beads kit (GE Healthcare) and a Thermal iCycler (Bio-Rad). The PCR primers used were the PRK341F (5′-CCTAYGGGRBGCAACAG-3′) and the PRK806R (5′-GGACTACNNGGGTATCTAAT-3′)^31,32^. PCR was performed as follows: an initial denaturation at 95 °C for 5 min, 35 cycles of denaturation at 95 °C for 30 s, annealing at 50 °C for 30 s and elongation at 72 °C for 70 s, and a final elongation at 72 °C for of 5 min. PCR products were visualised on 1% agarose gels stained with gel red using a GelDoc (Bio-Rad) and the Quantity One 4.1 software. Amplicon purification and Sanger sequencing were performed by the company Eurofins Genomics. Electropherograms were assembled using Geneious Prime (v2019.1.1). Sequences were manually corrected and trimmed, and BLASTn searches were performed against public databases for identification and comparison with 16S rRNA-based identification. Sequences and strain identifications of the three bacteria were submitted on GenBank.

#### Attempted inductions of the SKin Ulceration Disease in healthy *Holothuria scabra* sea cucumbers

A qualitative *pilot experiment* was first performed to test a potential induction of the SKUD in healthy *H. scabra* sea cucumbers via a direct contact with SKUD-affected sea cucumbers. Then, quantitative experiments were performed to test the potential pathogenicity of the three bacterial strains isolated from SKUD ulcers (see above “Isolation of bacteria present in the skin ulcerations of *Holothuria scabra* and molecular identification of bacterium taxa” section). For that, three different protocols have been used: (*i*) inoculation on the dorsal integument (*Experiment 1*), (*ii*) intracoelomic injection (*Experiment 2*), (*iii*) forced ingestion in the foregut of the sea cucumber (*Experiment 3*).

In the *pilot experiment*, 20 healthy adult *H. scabra* sea cucumbers were inoculated with grinded SKUD ulcers from sick individuals. For that, the ulcers from five sick sea cucumbers at stages IV (see “Results” “Symptoms of SKUD-affected *Holothuria scabra* sea cucumbers in the southwest of Madagascar” section for stage description) were milled using a manual food mill washed beforehand with bleach and rinsed with filtered seawater. The fluid obtained was directly applied on the dorsal integument of the sea cucumbers. Prior to inoculation, scarifications were manually performed on the dorsal integument of 10 individuals of *H. scabra.* For that purpose, sterile razor blades were used to perform five small superficial wounds on the dorsal integument of the healthy sea cucumbers (i.e. 1 cm long, 0.5 cm wide). In total, 20 inoculated sea cucumbers (i.e. ten scarified, ten non-scarified) were maintained in aerated 300 L-aquaria for ten days.

In *Experiment 1,* scarifications made on three healthy individuals were inoculated by one of the three isolated bacteria (see “Material and Methods” “Isolation of bacteria present in the skin ulcerations of *Holothuria scabra* and molecular identification of bacterium taxa section”, “Results” "Identification of isolated bacteria and results of infection test assays on the sea cucumber *Holothuria scabra*"). First, the sea cucumbers were scarified with a razor blade. For each tested isolated bacterium, bacterial colonies were swabbed with a sterile cotton and inoculated (i.e. brushed) on the wounds (i.e. scarifications) present on the dorsal integument of three sea cucumbers. After inoculation, sea cucumbers were placed in an aerated aquarium. As a negative control, scarification performed on three healthy individuals were inoculated with sterile culture medium (without bacteria).

Two additional controls were performed to evaluate the effect of the artificial scarifications. Five SKUD-affected sea cucumbers (stage II–III, see “Results” “Symptoms of SKUD-affected *Holothuria scabra* sea cucumbers in the southwest of Madagascar” section for stage description) were scarified with a razor blade. The evolution of the scarifications, but also, of their initial ulcers was followed daily for six days. In parallel, five healthy sea cucumber were scarified with a razor blade, and the evolution of the scarifications was followed daily for six days.

The *Experiment 2* consisted of an intracoelomic injection of 5 mL of a solution of bacteria in seawater (the same three bacterium species obtained before were tested individually). Obtaining the same concentration at each assay for the three injected bacterial cultures was made possible by the use of a spectrophotometer. Bacteria grown on solid medium were collected using a sterile cotton swab and resuspended to 0.22 µm filtered seawater. The optical density (OD) was then measured at 600 nm. An arbitrary DO value of 0.360 was chosen. This corresponded to an approximate concentration of 3 × 10^10^ bacteria per mL of solution (Agilent Technologies, 2017) or 15 × 10^10^ bacteria per 5 mL of injection. For each treatment, the experiment was performed on three individuals. As a negative control, sterile seawater was injected in three additional the sea cucumbers.

The *Experiment 3* consisted of a forced ingestion (i.e. using a plastic syringe) of 5 mL of a seawater solution containing bacteria in the foregut of the sea cucumbers. Again, a bacterial concentration of 3 × 10^10^ bacteria per mL of solution (Agilent Technologies, 2017) or 15 × 10^10^ bacteria per 5 mL of ingestion were used. For each treatment, the experiment was performed on three individuals. As a negative control, sterile seawater was used for forced ingestion in three additional the sea cucumbers.

For *Experiments 1, 2* and *3*, a daily monitoring of sea cucumbers was carried out for six days to observe the potential emergence of new ulcers, the surface increase or decrease of initial scarifications or any other symptoms. Sea cucumbers of around 350 g were used. Measurements of the dorsal integument ulceration surface (i.e. scarifications and ulcers) was performed using ImageJ^33,34^ and relative measurements are presented with the total dorsal integument surface considered as 100%.

All figures presented in the manuscript were edited using Adobe Illustrator 2020 (v24.3.0).

## Results

### Symptoms of SKUD-affected *Holothuria scabra* sea cucumbers in the southwest of Madagascar

**Figure 2:**
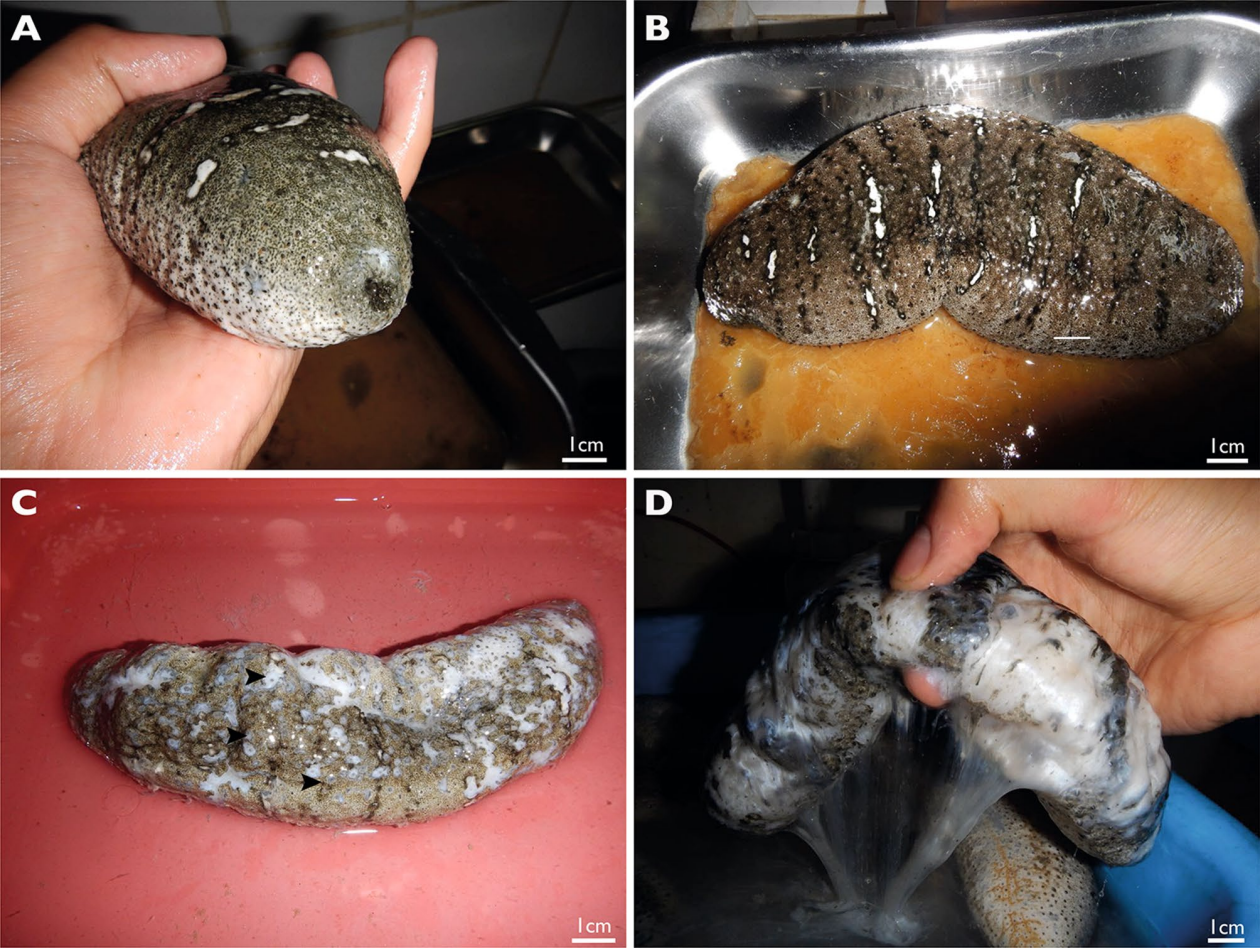
Phenotype characterisation of SKUD stages in *H. scabra*. (**A**) Stage I, (**B**) Stage II, (**C**) Stage III, (**D**) Stage IV.

Based on external observations of SKUD-affected *H. scabra* individuals, four stages were defined to characterise the level of progress of the disease (Fig. 2). Individuals at stage I of the disease have one or multiple small white lesions of no more than 0.5 cm in diameter around the cloaca, the mouth or both. The presence of white lesions all around the body, as well as the surface increasing of existing lesions (> 0.5 cm in diameter), characterise individuals in stage II of the disease. The mouth and cloaca are more severely affected by large ulcerations. Stage III individuals are characterised by a body covered by ulcerations present on the dorsal (i.e. bivium) and ventral (i.e. trivium) integument. The ulcers are several centimetres in diameter and may release a whitish-transparent mucus. Body wall perforations are present at the end of this stage connecting the coelom and the external environment. Eviscerations are also observed in Stage III. Stage IV individuals have degraded body where holes may release internal organs and whitish mucus. At this stage, vital signs of the sea cucumber are not recognizable: there is no cloacal respiration and no body wall contraction upon mechanical stimulation.

### The occurrence of the SKin Ulceration Disease (SKUD) in IOT sea pens (Toliara, Madagascar)

Between January 2016 and May 2017, at least 320,000 (August 2016) and up to 1,145,000 (May 2017) individuals of *H. scabra* were maintained in the sea pens of Belaza (Toliara, Madagascar) (IOT data). The increasing number of individuals in sea pens comes from the constant supply of the IOT nursery which provides young individuals (6 cm-long, 15 g). This number is varying from one month to another depending on the success of the pre-growing phase.

**Figure 3:**
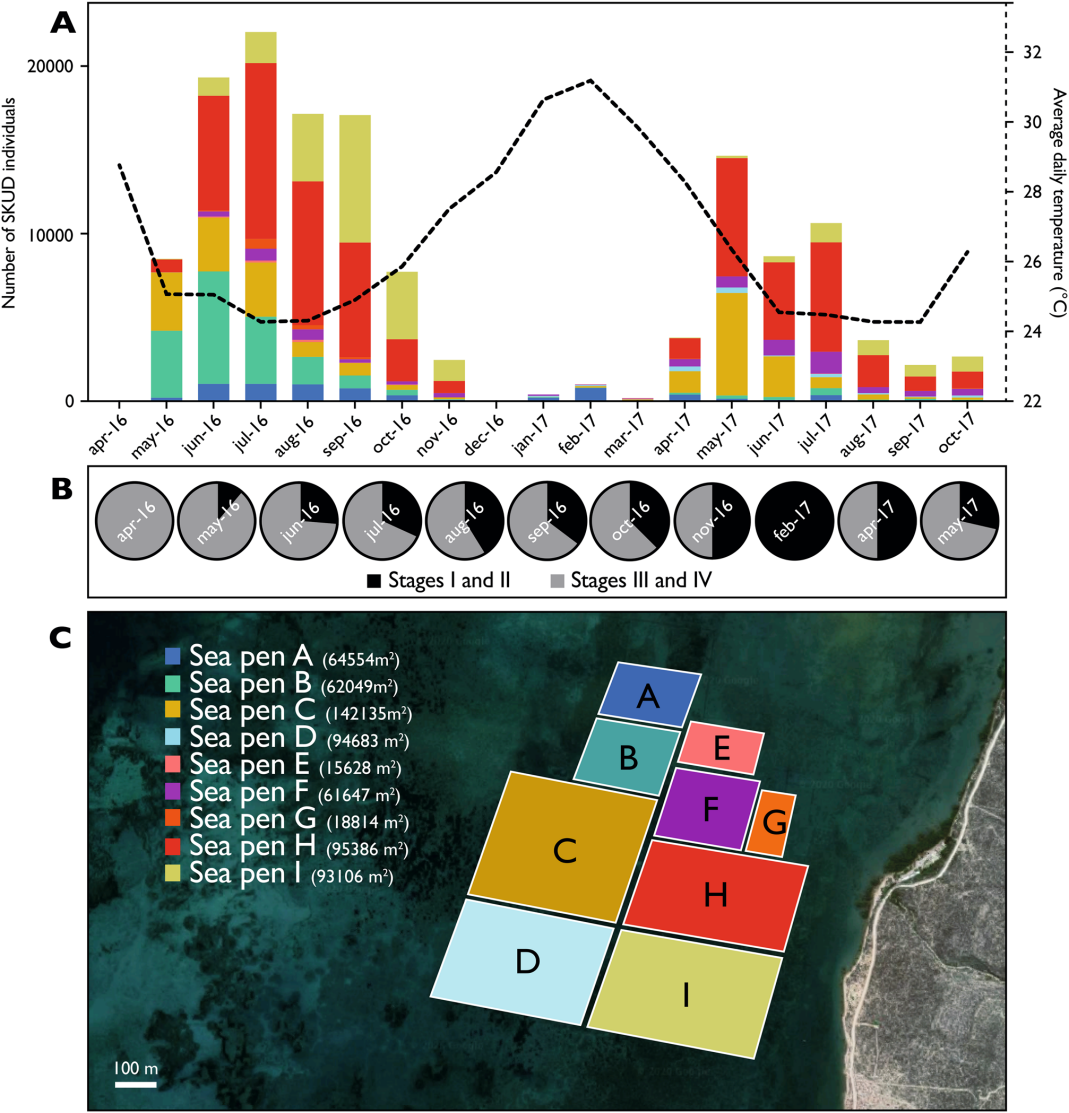
(**A**) Distribution of SKUD occurrence within IOT pens (Belaza, Toliara, Madagascar) between April 2016 and October 2017. The figure shows the monthly number of total sick *Holothuria scabra* individuals recorded in different IOT pens. The colour-code corresponds to the different pens that were investigated. The dotted line shows the evolution of average daytime temperatures measured in IOT pens (surface water). (**B**) Proportion of stages I/II and stages III/IV SKUD-affected *Holothuria scabra* sea cucumbers during selected months (see Fig. 2 for the description of the stages). (**C**) Organisation of IOT pens investigated in the present study. The map was obtained from Google map (https://www.google.be/maps, accessed in May 2020, Images 2020 Maxar Technologies, Images 2020 CNES/Airbus).

Although some sick individuals were punctually observed in the sea pens, it was in April 2016 that the first large number of affected individuals was detected (i.e. 52 sick individuals). The month after, in May 2016, the number of diseased sea cucumbers exploded (i.e. 8,454 sick individuals). A census of SKUD-affected individuals was then performed monthly in sea pens from April 2016 to October 2017 (Fig. 3A). The disease was epidemic and reached a peak in July 2016 with 21,891 sick individuals. The disease was intense between June and September, it decreased in intensity in October–November 2016 and disappeared in December 2016. In 2017, the disease affected 1036 individuals in February but reappeared in April (i.e. 3779) and peaked in May when 14,686 individuals were affected. The intensity of the epidemic then decreased with some 2711 sick individuals collected in October. In total, over 17 months from January 2016 to October 2017, 143,428 individuals were sick. The proportion of stages (I/II and III/IV) was evaluated during 11 months of the investigated period. From the emergence of the first cases, a high proportion of advanced SKUD-stages is observed. A higher proportion of early SKUD-stages was, however, observed during the hot season (e.g. February 2017) (Fig. 3B).

In parallel, daytime water temperatures were recorded monthly in sea pens. Average daytime temperatures varied from 24.3 in September 2017 (with a minimum temperature measured at 23.6 °C) to 31.4 °C in March 2016 (with a maximum temperature measured at 32.3 °C). In the epidemic months in 2016 (June to September), the temperature varied between 23.34 and 25.88 °C. During the epidemic months in 2017 (May to July), the temperature varied between 23.25 and 26.87 °C. When the number of diseased individuals was close to zero, in December 2016, January 2017 and March 2017, the average daytime temperatures were 28.6, 30.6 and 29.8 °C, respectively (Fig. 3A). The occurrence of SKUD-affected sea cucumber was evaluated in nine sea pens (represented in the Fig. 3C).

### Histology and fine structure of skin ulcerations in *Holothuria scabra*

**Figure 4:**
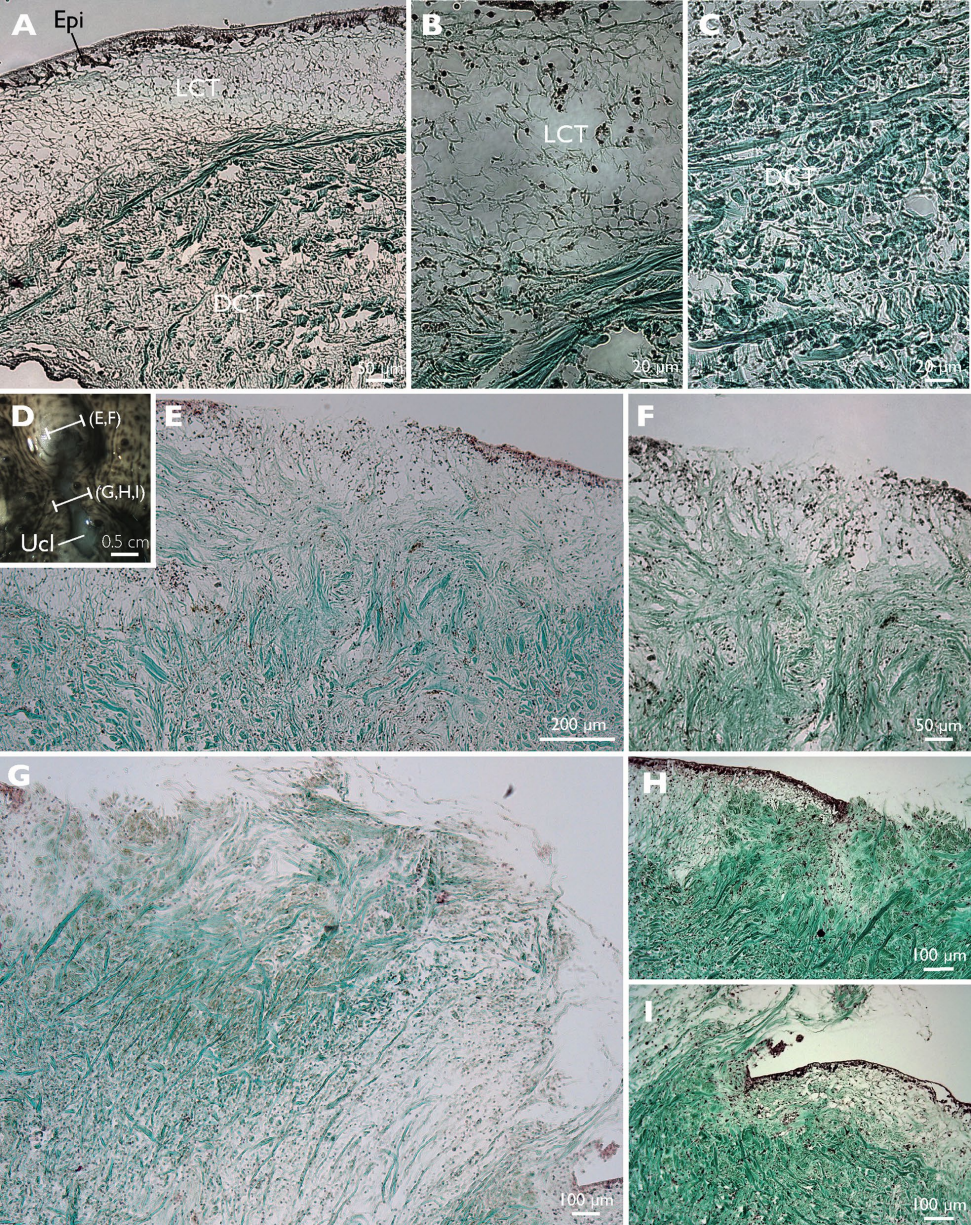
Histology of healthy and SKUD-affected *Holothuria scabra* sea cucumber integuments. (**A**–**C**), (**E**–**I**) Masson’s Trichrome staining. (**A**–**C**) healthy integument, (**D**–**I**) SKUD-affected integument. Legend: *Epi* epidermis, *LCT* loose connective tissue, *DCT* dense connective tissue, *Ucl* ulcer.

On histological sections of healthy tissues, an epidermis of 50 µm in thickness covers a dermis with a thickness of several tens of millimeters. The dermis is made of an outer part of loose connective tissue (LCT) of 250 µm in thickness and an inner dense connective tissue that reaches the mesothelium bordering the general body cavity (i.e. the somatocoel) (Fig. 4A–C). Spaces, corresponding to the ossicles dissolved by the preparation method, are disseminated in the loose connective tissue. In the dense connective tissue, collagen fibres appear in bundles that are about 5 μm thick (Fig. 4C). These bundles have various orientations, some appear parallel to the surface of the body, others oblique or perpendicular.

Histological sections of ulcerations were performed at the rim (Fig. 4D–F) and in the middle of a lesion (Fig. 4D,G–I). At the start of the lesion, the total disappearance of the epidermis is observed over a length of 500 μm (Fig. 4E,F). Under the place where the epidermis is absent, the loose connective tissue is invaded by collagen bundles coming from the dense connective tissue. These bundles form a kind of "T" shaped structure whose upper part tends to close the opening left by the epidermal disappearance. In the middle of the lesion, the collagen bundles coming from the dense connective tissue form a plug which protrudes from the body surface by several hundreds of microns (Fig. 4G–I). At the edge of the lesion, under the epidermis, the loose connective tissue is reduced, not measuring more than 100 μm, and is replaced by the collagen fibres from the dense connective tissue. In an ulceration context, the white colour of the lesions appears to be due to the exposure of the connective tissue that follows the destruction of the cuticle, the epidermis and the upper part of the connective tissue.

**Figure 5:**
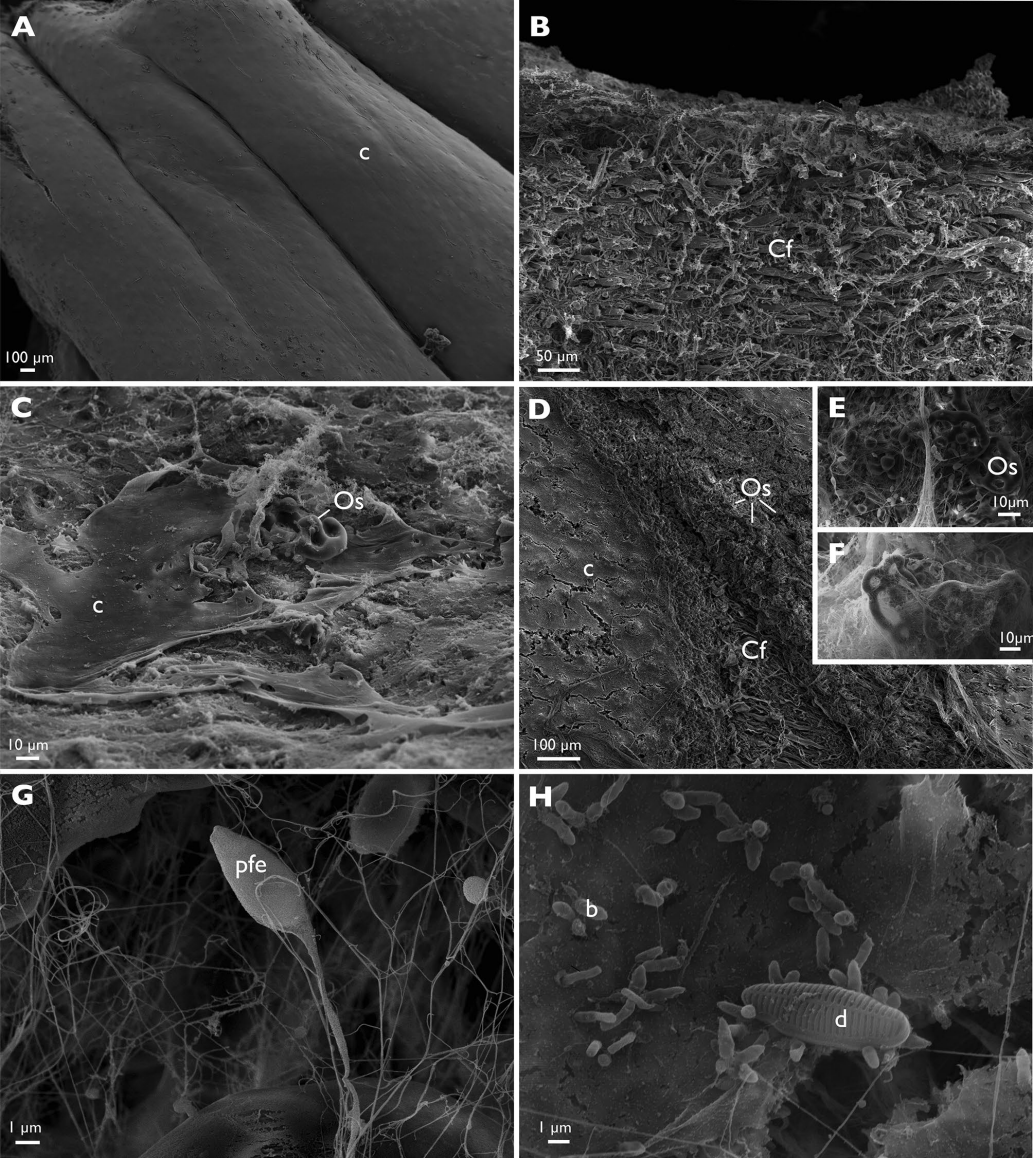
The fine external structure of healthy and SKUD-affected *Holothuria scabra* sea cucumber integument. (**A**,**B**) healthy integument, (**A**) external surface of the integument, (**B**) connective tissue present under the epidermis (transverse section). (**C**–**H**) SKUD-affected integument. (**C**–**H**) external surface of the ulcerated integument. (**E**–**H**) Ossicles, bacteria and diatoms visible at the ulcer surface. Legend: *b* bacteria, *c* cuticle, *Cf* collagen fiber, *d* diatom, *pfe* predicted flagellated eukaryotes, *Os* ossicle.

In scanning electron microscopy, the integument surface of healthy animals appears smooth (Fig. 5A) with an underlying fibrous connective tissue (Fig. 5B). Near the lesions, the surface of the sick animals is rough (Fig. 5C). At the level of the lesions where the epidermis has disappeared, the collagen fibres as well as the ossicles are now visible (Fig. 5C–G). Microorganisms are observed in and around the lesions such as rod-shaped non-flagellated bacteria that are around 2 μm long. Their concentration near the lesions is high, of the order of several dozen per 100 μm^2^ of the surface observed. A fibrillar material of a few nanometer in thickness and about 10 μm in length is also observed and can be interpreted as protein fibers originating from the degraded connective tissue or from “filamentous production” of microorganisms (Fig. 5G–H). Potential flagellated eukaryotes (Fig. 5G) and diatoms have also been observed but in small numbers (Fig. 5H).

**Figure 6:**
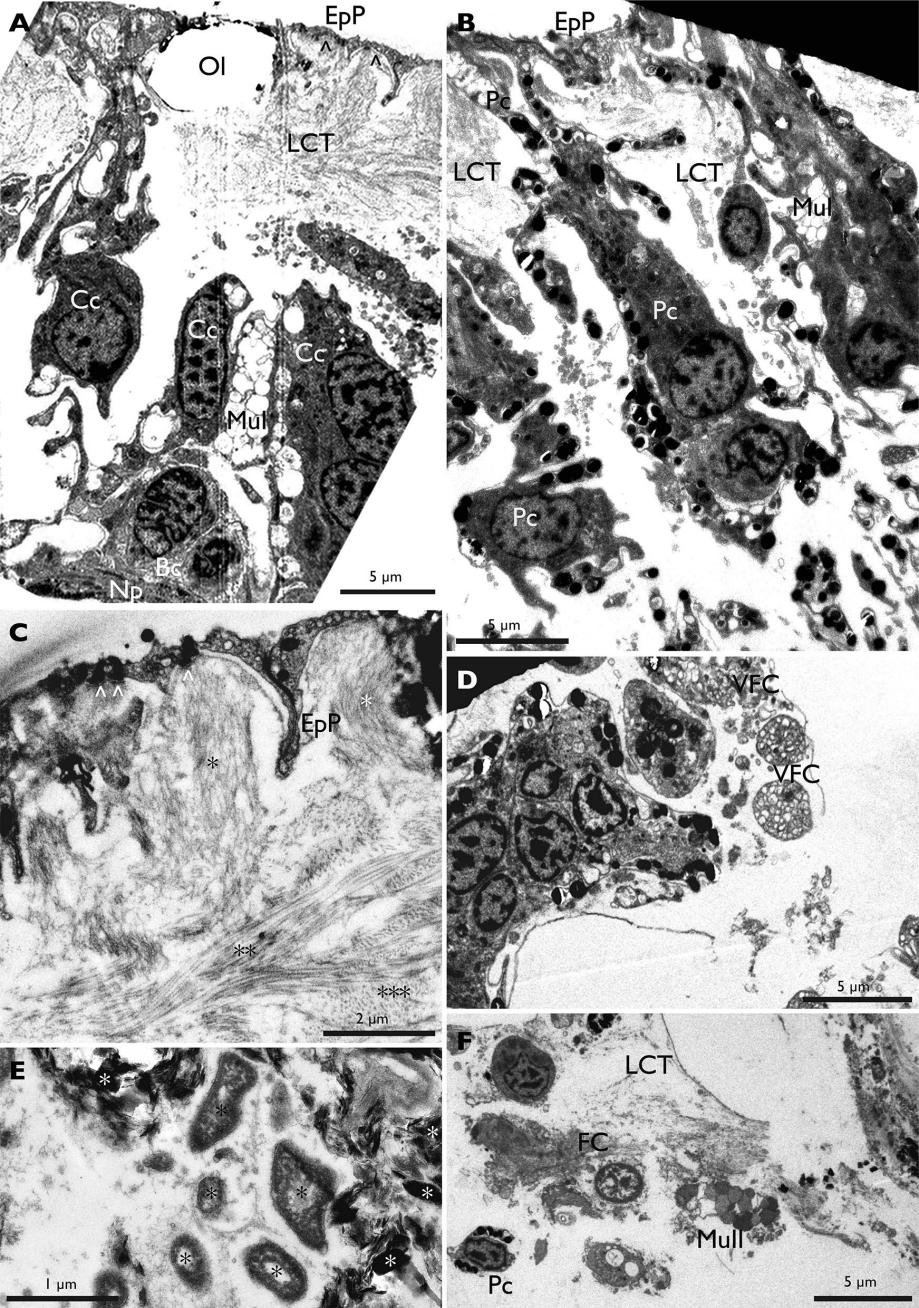
TEM observations of the dorsal integument of healthy and SKUD-affected *Holothuria scabra*. (**A**–**C**) healthy integument; (**D**–**F**) SKUD-affected integument. The upper portion of the body wall showing the thin cell processes of covering cells and the loose connective tissue with the perikaria of basal cells, covering cells, type I mucocytes (**A**; arrows show the thin epidermal processes and asterisks underline the various collagen fibre orientations); pigment cells (**B**); and a detailed view of first micrometres from the surface showing the nanometric epidermal covering and the orientation of collagen fibres (**C**; arrows show the hemidesmosomes). SKUD-affected integument shows degrading cell clusters with vacuolated fragmented cells (**D**); degrading cells into the loose connective tissue (**E**); and invading bacteria (black asterisks) and degrading cell material with electron-dense crystalline bodies (white asterisks) (**F**). Legend: *Bc* basal cell, *Cc* covering cell, *EpP* epidermal process, *LCT* loose connective tissue, *MuI* mucocyte cell of type I, *MuII* mucocyte cell of type II, *Np* nerve process, *Ol* ossicle lacunae, space of dissolved ossicle, *Pc* pigment cell, *VFC* vacuolated fragmented cell.

**Figure 7:**
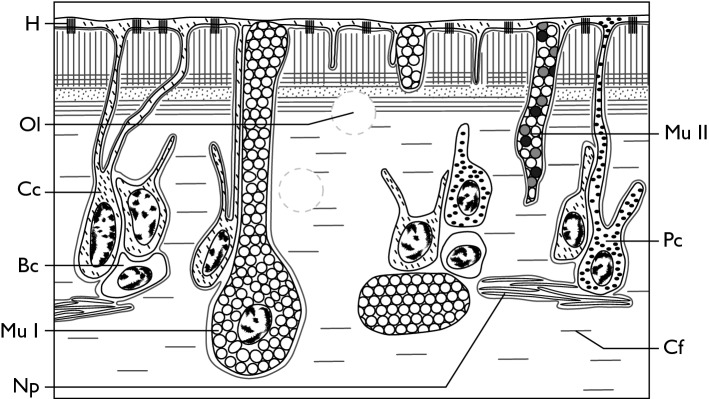
Schematic view of the fine structure of the *Holothuria scabra* dorsal integument. This schematic representation is based on TEM observations. Legend: *Bs* basal cell, *Cc* covering cell, *Cf* collagen fiber, *H* hemidesmosome, *Mu I* Type I mucocyte, *Mu II* Type II mucocyte, *Np* nerve process, *Ol* ossicle lacunae, *Pc* pigment cell.

Transmission electron microscopy allowed us to understand the fine structure of the healthy epidermis of the dorsal integument in *H. scabra* and the underlying loose connective tissue as well as the changes in these tissue layers induced by ulcerations. The surface of the epidermis is mainly made up of very fine cellular processes which emanate from the covering cells, the perikaria of these cells being located deeper into the loose connective tissue. Due to this arrangement, the general epidermis of the dorsal integument appears to consist of T-shaped structures, the horizontal part forming the surface of the body and being composed of the thin cellular processes of the covering cells and the vertical part (perpendicular to the surface of the body) being formed by the perikarya of four cell types (cell names were given according to previous reference literature^35^): covering cells (CC), pigment cells (PC), two types of mucocyte cells (Mu I and Mu II) and basal cells (BC) (Fig. 6, Fig. 7 for a schematic representation). Pigment cells and mucocytes both contact the body surface. These cells form clusters sunk about 50 μm into the loose connective matrix and are spaced from each other by 10 to 50 μm. The perikaryon of covering cells measures *ca.* 7 μm in diameter and includes a nucleus surrounded by a fine cytoplasmic fringe with a little charge of cellular organelles. From their apex leave several thin cellular processes of the order of 1 μm thick which reach the surface. The epidermal surface formed by the cellular processes that cover the dorsal integument measures from 100 nm to 1 μm in thickness at most. All of these very fine cellular processes are underlined by a basal lamina through which hemidesmosomes cling to collagen fibres from the loose connective tissue. These collagen fibres form stacks of variable orientations: those which are attached to the basal membrane are perpendicular to the surface, the underlying ones are parallel to the surface and form successive layers of variable orientation.

Type I mucocyte cells have a large perikaryon of *ca.* 15 µm in diameter which is found up to 50 µm below the epidermal surface. Their cytoplasm is filled with electron-lucent secretory vesicles of *ca* 1 to 2 µm in diameter. Type II mucocyte are very similar but their secretory vesicles are filled with clear fibrillar material. In both mucocyte types, these vesicles are brought to the surface by cellular processes. The pigment cells are thinner, around 7 µm at the perikaryon, and their cytoplasmic fringe contains small dense electron granules which are also present in the cytoplasmic processes that connect to the most external part of the epidermis. The basal cells are small spherical cells about 7 µm in diameter, without cell process, found in the loose connective where nerve processes of the plexus are also observed.

In TEM, the loose connective tissue includes many empty spaces which correspond to the location of the ossicles that have been dissolved. The cuticle is also not visible because it disappeared in the glutaraldehyde fixation process.

In ulcers (Fig. 6D–F), the degrading epidermal cells form clusters where the cell types are very difficult to distinguish. Associated with these clusters, we found bacteria located outside of the degraded cells. These bacteria are the only microorganisms that could be identified there. Furthermore, the structure of the collagen fibres of the connective tissue is also altered and these fibres no longer overlap in separate layers.

### Metagenomic characterisation of the SKUD-affected dorsal integument of *Holothuria scabra*

Four samples, coming from two SKUD-affected *Holothuria scabra* sea cucumbers (i.e. individual 1—replicate 1 and individual 2—replicate 2), were analysed (i.e. two ulcer samples, two healthy integument samples). Between 45,318,062 and 68,361,010 raw reads were generated for each sample (Table 2). The experiment set-up that was used during our study (i.e. dissection of healthy and ulcerated integument fragments) produced a large quantity of host reads (i.e. sea-cucumber) in our genomic dataset. No prior selection of the microorganisms present on the integument of sea cucumbers has been attempted to avoid any potential selection or bias. The MG-RAST functional gene sequence analysis showed that Eukaryota was indeed the dominant domain in all samples (between 92.69 and 97.10%) which mainly corresponds to the sea cucumber integument tissue (Supplementary Fig. 1). Bacteria were the second dominant domain in all samples representing 7.25% and 2.85% for skin ulcer samples of the two individuals and 6.91% and 5.70% for the healthy integument samples of in the two same individuals. Viruses and Archaea represented less than 0.03% in all samples.

**Table 2:** Raw and filtered reads and global annotation using the MG-RAST pipeline. Total Reads: Total number of sequence reads analysed for each sample. Raw reads were submitted on NCBI under the Bioproject PRJNA634538. Legend: SKUD integument: dorsal integument affected by the SKUD (i.e. ulcer); Healthy integument: healthy dorsal integument of a SKUD-affected individual; Ind.: individual; Rep.: replicate.

Sample name	Total reads	Filtered reads	Ribosomal RNA genes	Predicted proteins with known functions	Predicted proteins with unknown function
Healthy integument Ind 1/Rep 1	68,361,010	59,619,253	230,561	4,785,315	32,344,653
SKUD integument Ind. 1/Rep. 1	62,891,886	57,144,571	208,367	4,235,173	31,769,230
Healthy integument Ind 2/Rep 2	67,074,290	58,470,267	231,046	4,605,557	32,238,624
SKUD integument Ind 2/Rep 2	45,318,062	38,863,762	138,689	3,143,388	21,734,797

**Figure 8:**
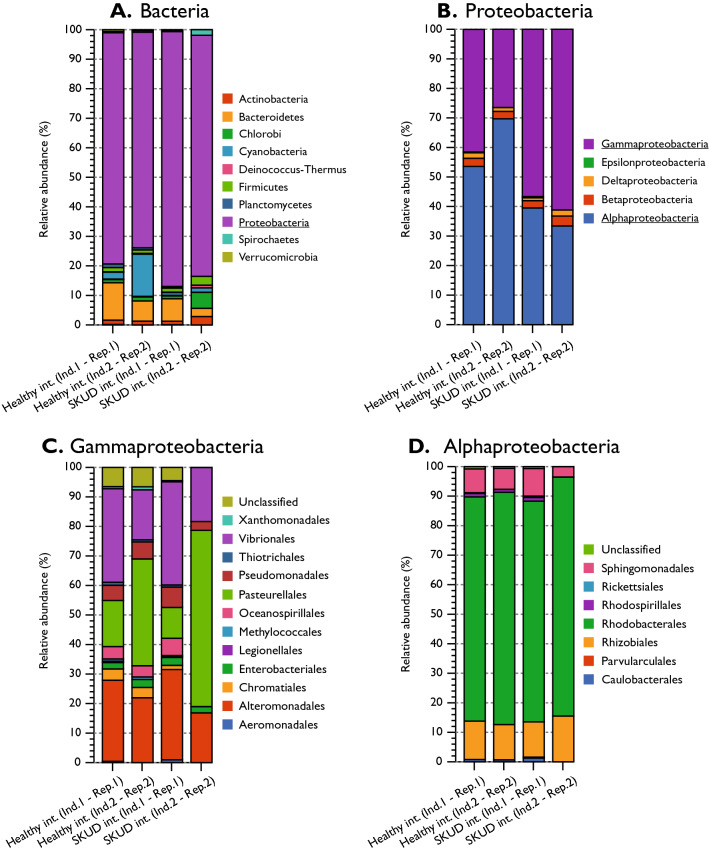
The relative proportion of Bacteria within healthy integument and ulcerated integument of two SKUD-affected *Holothuria scabra* sea cucumber individuals. (**A**) Bacteria, (**B**) Proteobacteria, Gammaproteobacteria, (**D**) Alphaproteobacteria. (MG-RAST parameter: E-value > e^−10^, Percentage Identity > 70%, Alignment length > 15, Minimum Abundance = 50, Representative hits, database: RefSeq). See supplementary Table S1 for statistical analyses.

In all four samples, Proteobacteria were the most represented bacteria and this proportion was significantly higher in skin ulcer samples (86.33 and 81.63% in SKUD integument individuals 1 and 2, 78.34 and 72.99% in healthy integument individuals 1 and 2, respectively) (Fig. 8A). Proteobacteria were the only group with a higher proportion in both ulcer samples (SKUD integument individuals 1 and 2) and the differences were statistically significant for both individuals (z-test, *P* value < 0.05; Supplementary Table S1 for statistical analyses). In proportion, Proteobacteria were directly followed by Bacteroidetes in all samples excepted for the healthy integument of the individual 2 where a relatively high proportion of Cyanobacteria was detected (i.e. 14.29%). Other represented bacterium phyla were Chlorobi, Firmicutes, Deinococcus-Thermus, Planctomycetes, Spirochaetes and Verrucomicrobia. No striking differences were observed, for these taxa between, ulcerated and healthy integuments. Within Proteobacteria, a significantly higher proportion of Gammaproteobacteria was observed for SKUD integument individuals 1 and 2 (56.62 and 61.19%, respectively, against 41.48 and 26.48% for healthy integument individuals 1 and 2, respectively; z-test, *p*-value < 0.05). A statistically significant lower proportion of Alphaproteobacteria was observed in the two skin ulcer samples (Fig. 8.B, z-test, *p*-value < 0.05). All other Proteobacteria clades (i.e. Betaproteobacteria, Deltaproteobacteria, …) have relatively constant proportions within all samples (Fig. 8B). Within the Gammaproteobacteria, Pasteurellales, Vibrionales and Alteromonadales constitute important clades, in terms of proportions, for all samples (Fig. 8C). Only Vibrionales, were found in higher proportion in both ulcer samples. The difference was statistically significant for the case of the individual 1 (SKUD int. compared to the healthy int; z-test, *p* value < 0.0001). No specific pattern, however, was observed for the proportions of Gammaproteobacteria and Alphaproteobacteria orders (Fig. 8C,D). For the Alphaproteobacteria, largely represented clades were “unclassified Alphaproteobacteria”, Rhizobiales and Sphingomonadales with relatively constant proportions within all samples.

In parallel, proportions of all bacterium families and genera were investigated (Figs. S2 and S3). This indicates that Vibrionaceae is the most represented bacteria family within all samples but also that the proportion of detected Vibrionaceae is higher in both ulcer samples (z-test, *p*-values < 0.0001 confirming the tendency observed with the proportions of Vibrionales).

The genus proportions were illustrated for descriptive purpose and it confirms that *Vibrio* is the most represented bacterial genus within all samples and its proportions are higher in ulcer samples. Previous studies indicated the potential importance of *Vibrio* and associated bacteria (Vibrionales, e.g. *Photobacterium*) in the potential emergence of SKin Ulceration Disease in sea-cucumbers^3,6,7,12–14^. The predicted species proportions within the Vibrionale order was investigated for descriptive purpose (Fig. S4). Predicted *Vibrio parahaemolyticus* appeared to be the most common bacterium found within all 4 samples.

The proportions of potential non-metazoan eukaryotes were also visualised as ‘protozoans’ and other unicellular eukaryotes that may be involved in marine invertebrate diseases. Considering the paraphyletic status of these organisms, the compartment “Eukaryota without Metazoans, Fungi and Streptophyta” (including, in our dataset, Apicomplexa, Chromerida, Euglenida, Phaeophyceae, Xanthophyceae) was used for the read selection (Fig. S5). At the phylum level, the proportions of the compartments Apicomplexa, Chlorophyta (i.e. green algae) and “unclassified (derived from Eukaryota)” were significantly higher in SKUD integument samples of both individuals (Supplementary Figure S5, Supplementary Table S2 for statistical analyses). More specifically, at the family level, the proportions of the compartments Cryptosporidiidae (Coccidia, Apicomplexa), “unclassified” (Haemosporida, Apicomplexa), “unclassified” (Prasinophyceae, Chlorophyta), Geminigeraceae (Cryptophyta, “unclassified (derived from Eukaryota)”), Perkinsidae (Perkinsida, “unclassified (derived from Eukaryota)”), Parameciidae (Peniculida, “unclassified (derived from Eukaryota)”) and Codonosigidae (Choanoflagellida, “unclassified (derived from Eukaryota)”) were significantly higher in SKUD integument samples of both individuals.

The proportion of represented Fungi families was also investigated for the descriptive purpose (Fig. S6, Supplementary Table S2 for statistical analyses). Sordariaceae and Tricholomaceae (Ascomycota) were the most represented fungi families in all samples. In terms of family proportions, no significant differences between ulcer samples and healthy integument samples were observed.

The proportion of virus orders and families was investigated for descriptive purpose (Fig. S7, Supplementary Table S2 for statistical analyses). No significant differences between ulcer samples and healthy integument samples were observed. However, no virus particle enrichment was performed before the DNA extractions. The two most represented virus families are Herpesviridae (Order Herpesvirales) and Poxviridae (Order “unclassified” in MG-rast analysis pipeline) in all samples.

### Identification of isolated bacteria and results of infection test assays on the sea cucumber *Holothuria scabra*

#### Pilot experiment: grinding SKUD ulcers and inoculation on the dorsal integument of healthy sea cucumbers

**Figure 9:**
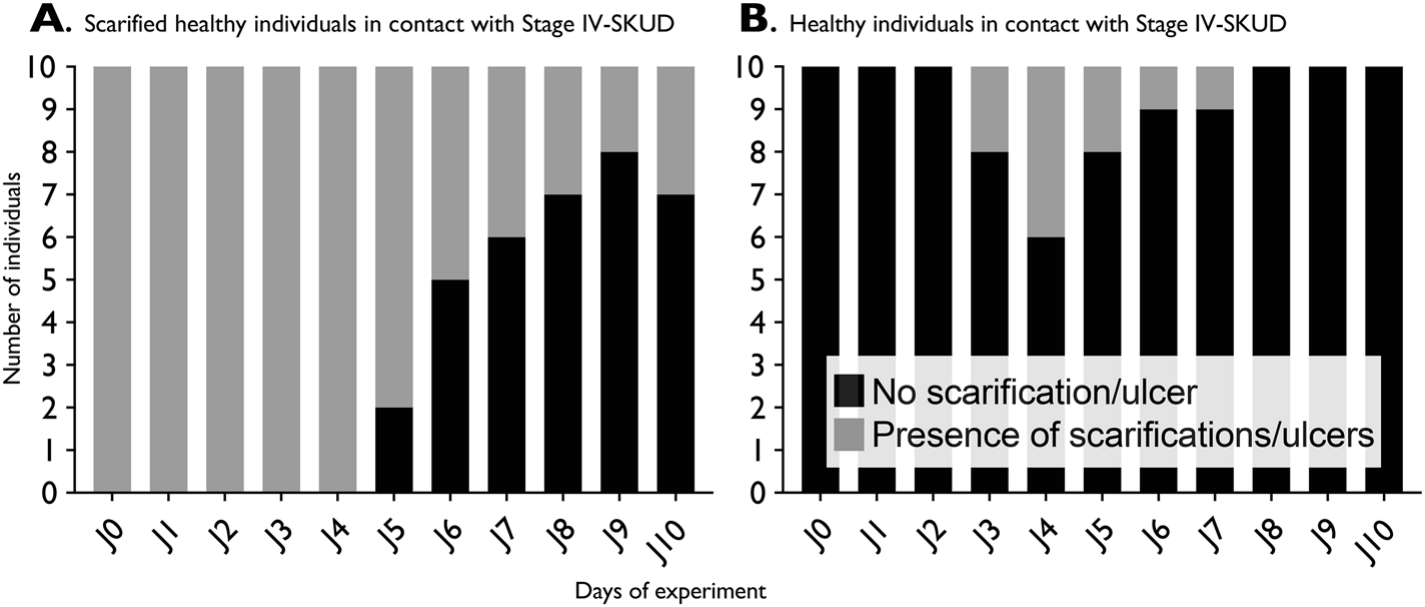
Attempted induction of SKUD in *Holothuria scabra*: qualitative pilot experiment performed to test a potential induction of the SKUD via a direct contact between healthy sea cucumbers and SKUD-affected sea cucumbers. (**A**) Inoculation of 10 scarified healthy individuals. (**B**) Inoculation of 10 non-scarified healthy individuals. Observations were performed for 10 days.

A qualitative pilot experiment was performed to test a potential induction of the SKUD in *H. scabra* via a direct contact between healthy sea cucumbers and SKUD-affected sea cucumbers. In total, 20 individuals were inoculated with grinded SKUD ulcers (i.e. 10 scarified, 10 non-scarified) and maintained in aquaria for 10 days. For scarified individuals, the scarification/ulceration surface decreased significantly during the experiment (Fig. 9A). The scarifications were observed during the first 4 days in all tested individuals. After six days, 50% of the sea cucumbers totally recovered without any additional symptoms. After ten days, the scarifications were only slightly visible in three individuals.

For non-scarified individuals, we observed the presence of small ulcers in four individuals after four days (Fig. 9B). However, these ulcers all healed before the 8^th^ day. No other symptoms and no mortality were observed during the experiment.

#### Integument scarification and Bacterium inoculation on scarified dorsal integument (Experiment 1)

**Figure 10:**
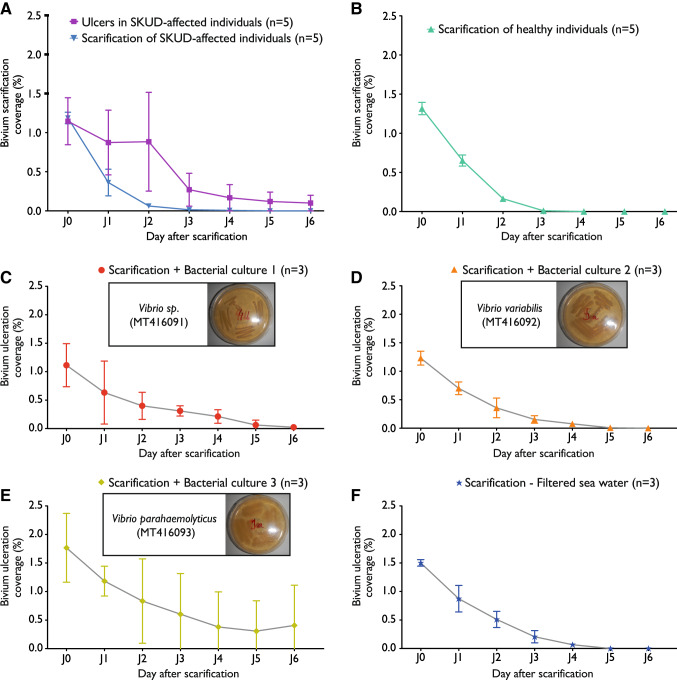
Attempted induction of SKUD in *Holothuria scabra* via the inoculation of cultured bacteria on the dorsal integument: three cultured bacteria, isolated from SKUD ulcers, were inoculated on artificial scarifications performed on the dorsal integument of the sea cucumbers. (**A**) Integument scarification/ulceration coverage observed for SKUD-ulcers (purple line) and artificial scarifications (blue line) in SKUD-affected sea cucumbers (controls). (**B**) Integument scarification/ulceration coverage observed in scarified healthy sea cucumbers (controls). (**C**) Integument scarification/ulceration coverage observed after inoculation of *Vibrio* sp. SKUD-MD-20 C1 (culture 1). (**D**) Integument scarification/ulceration coverage observed after inoculation of *Vibrio* *variabilis* SKUD-MD-20 C2 (culture 2). (**E**) Integument scarification/ulceration coverage observed after inoculation of *Vibrio parahaemolyticus* SKUD-MD-20 C3 (culture 3) (**F**) Integument scarification/ulceration coverage observed after inoculation of filtered sea-water (negative controls).

To test a potential bacterial induction of the SKUD in *H. scabra*, three bacterial strains isolated from *H. scabra* ulcers were cultured and identified using 16S rRNA gene amplicon sequencing. Isolated bacteria were inoculated within the scarifications. Controls were performed to (i) reveal the effect of integument scarification and observe the natural recovery of integument ulcers in SKUD-affected sea cucumbers maintained in controlled conditions (Fig. 10A) and to (ii) reveal the effect of integument scarification in healthy sea cucumbers (Fig. 10B). The first bacterial culture corresponds to a *Vibrio* sp. (culture 1, *Vibrio* sp. SKUD-MD-20 C1, GenBank: MT416091) (Fig. 10C). The second culture corresponds to a *Vibrio variabilis* (*Vibrio variabilis* SKUD-MD-20 C2*,* GenBank: MT416092) (Fig. 10D). The third culture corresponds to a *Vibrio parahaemolyticus* (*Vibrio parahaemolyticus* SKUD-MD-20 C3, GenBank: MT416093) (Fig. 10E). BLAST results of all three bacteria sequences are presented in the Supplementary Table S3.

Regarding the body wall inoculation, Fig. 10 shows the evolution during six days of the scarified surface of the nine sea cucumbers for which the body wall was inoculated with one of the three isolated bacteria (i.e. cultures 1, 2, 3) (Fig. 10C–E). As a negative control, the experiment was also performed by inoculating filtered sea water (Fig. 10F). On average, the scarified surface starts at 1.39% of the total body surface. After six days the ulceration surface was reduced to almost zero for *Vibrio sp.* and *V. variabilis* inoculation treatments (as well as for the controls) indicating that the natural recovery was apparently not affected. A higher variability was observed for individuals inoculated with *Vibrio parahaemolyticus* but the same general tendency is observed.

The three individuals inoculated with *Vibrio* sp. SKUD-MD-20 C1 (culture 1) saw new lesions that appeared at the level of the mouth or the cloaca. However, only one individual still had these lesions on the last day of the experiment. Yet, the three individuals inoculated with *Vibrio variabilis* SKUD-MD-20 C2 showed, in the end, no degradation of their integument. With *Vibrio parahaemolyticus* SKUD-MD-20 C3, one individual showed no degradation of the integument. Another eviscerated from the start and multiple lesions appeared on the dorsal integument and near the cloaca. Only one reduced lesion near the cloaca persisted until the end of the experiment. As for the last individual, it eviscerated from the first day of the experiment and its state of health went into decline during the experiment. The experiment resulted at the end by the appearance of large perforating lesions on the dorsal and ventral integuments. In the three control sea cucumbers, one contracted a lesion close to its cloacal opening on the third day, which resolved before the end of the experiment. The other individuals showed no degradation of their integument.

#### Bacterium intracoelomic injection (Experiment 2)

All of the individuals injected with predicted *Vibrio* sp. SKUD-MD-20 C1 (culture 1) or *Vibrio variabilis* SKUD-MD-20 C2 (culture 2) showed no degradation of their integument. Two individuals injected with *Vibrio parahaemolyticus* SKUD-MD-20 C3 (culture 3) have seen a deterioration of their health status. One individual saw the emergence of several whitish lesions on the ventral integument during the 6th day. A second individual died the same day. As for control, no individual has seen degradation of its integument. No evisceration took place during this experiment.

#### Bacterium ingestion (Experiment 3)

The three individuals having ingested *Vibrio* sp. SKUD-MD-20 C1 (culture 1) showed no degradation of their integument. One individual having ingested *Vibrio variabilis* SKUD-MD-20 C2 (culture 2) presented on the last day of the experiment a benign whitish lesion on bivium. The two other individuals showed no degradation of their integument. One individual having ingested *Vibrio parahaemolyticus* SKUD-MD-20 C3 (culture 3) experienced the appearance of an ulcer on the dorsal integument that resorbed in the last days. Other individuals showed no degradation of their integument, but one individual eviscerated from the first day of the experiment with no further weakening. In summary, 80% of the individuals tested in this experiment showed no integumentary damage after six days of observation.

## Discussion

In the scientific literature, deep integument ulceration leading to the death of sea cucumbers in farming have been described in Oceania (Australia), Africa (Madagascar), Asia (Indonesia, Malaysia, Vietnam, China) and Europe (Portugal) and it affects, at least, three species: *H. scabra, H. arguinensis* and *A. japonicus*^1–4^. Skin ulceration also affects other species that are investigated for aquaculture development trials. The authors of the present work have observed skin ulceration in *Bohadschia vitiensis* in Madagascar, *Isostichopus badionotus* in Colombia and *Holothuria forskali* in France which indicates that SKin Ulceration Diseases can be observed on sea cucumbers all over the world or at least on shallow water sea cucumbers in tropical and temperate regions (i.e. no skin ulceration has been reported on abyssal sea cucumbers or in species from the polar regions). So far, there is no nomenclature providing information on sea cucumber diseases where ulcerations are observed and it is generally referred to a single SKin Ulceration Disease regardless of where it occurs, and which species is affected. There are however many diseases which induce skin ulceration (in echinoderms in general, and in sea cucumbers in particular) and the integument ulceration should be considered as a symptom and not as a single disease in itself.

Overall, a disease can be congenital or acquired and when acquired, it can be caused by biotic (i.e. pathogens) or abiotic (i.e. environmental factors) causes. In the case of SKUDs, the factors which initiate these diseases may, in some cases, be biotic in other cases, abiotic. It would probably be useful to define an adequate nomenclature for these SKUDs and we propose to associate with the acronym of the disease (*SKUD*) an indication of the sea cucumber species affected by the first letters of the genus and the species (e.g. *Hs* for *Holothuria scabra*), an indication of the place of departure of the disease via ISO code 3166–2 (e.g. *MG* for Madagascar), the date of description of the disease (e.g. *20* for the year 2020), and, when known, an indication of the inducing agent represented by the first letter of the general taxon (b for bacteria and v for virus in currently known cases), a “*a*” if it is an abiotic inducing parameter or nothing if the inducing cause has not been precisely identified. Under this nomenclature, the disease described in this work will be designated under the name *SKUD Hs-MG-20.*

At present, four SKUDs described in the literature are diseases that have been re-induced by a single isolated pathogen: *Aj-CN-10b* has been induced by the bacterium *S. marisflavi*^18^, *Aj-CN-11b* by *V. splendidus*^13,14^, *Aj-CN-10b* by the bacteria *Pseudoalteromonas sp.*^5^ and *Aj-CN-10v* by an unidentified virus^5^. Also, other SKUDs have been induced using a consortium of bacteria^5,18^. One disease, the SKUD *Hs-MG-19a* was induced by a diet containing a large proportion of animal organic matter^17^. In the present work, it is highly probable that the initiation of the disease is not due to a pathogen or, at the very least, that if this disease is induced by a pathogen, that this latter is not found within the ulcerations. Indeed, this disease is not transmissible by contact: even when making scarifications and brushing them with the degraded material of the wounds of individuals affected by SKUD, these scarifications heal, and the very great majority of individuals are found unharmed after a few days. Also, three species of bacteria were isolated (i.e. *Vibrio* sp. SKUD-MD-20 C1, *Vibrio variabilis* SKUD-MD-20 C2*, Vibrio parahaemolyticus* SKUD-MD-20 C3) from SKUD ulcers and the brushing of scarifications by these bacteria were inefficient to induce the disease. Even though bacterial cultures are very selective, the combination of these results strongly suggests that sick wounds are unable to induce disease in healthy animals. Shotgun metagenomic results confirmed that Vibrionaceae (*Vibrio*, in particular) is one of the most abundant bacterial family detected on the skin of sick animals (i.e. on ulcers but also on healthy integument area). *Vibrio* spp*.* are widespread in the aquatic environment and are often found associated with various organisms ranging from plankton to animals^36^. In the last few years, the taxonomy knowledge of *Vibrio* has been improved, and many novel species have been described^37–39^. There are now over 100 formally described species in the genus. Some *Vibrio* species have been described to cause severe infections in humans and in many marine organisms (i.e. fish, crustaceans, molluscs and coral), but *Vibrio* bacteria are also considered to be part of the normal microbiota of marine invertebrates and fishes^36,40,41^. One of the isolated bacteria from ulcerations was identified as a species close to *Vibrio variabilis* which was first identified from the mucus of apparently healthy cnidarians *Palythoa caribaeorum*^37^. The second isolated bacterium from ulcerations was as a species close to *Vibrio parahaemolyticus* which is a curved, rod-shaped, Gram-negative bacterium found in brackish water, and which, when ingested, can causes gastrointestinal illness in humans. This bacterium is also known to cause diseases to marine invertebrates, including the acute hepatopancreatic necrosis disease (AHPND) which has been causing global losses to the shrimp farming industry^42^. In the second set of experiments, we inoculated these three bacteria in the digestive system (by food ingestion) and in the coelom (by injection at the level of the general cavity) of *H. scabra*. Apart from a slight induction of ulcerations caused by *V. parahaemolyticus*, the initiation of SKUD *Hs-MG-20* has not been conclusive.

Shotgun metagenomic analyses also highlighted a higher proportion of some unicellular eukaryotes with SKUD ulcerations. Some of these organism taxa have been reported to parasite or infest echinoderms like coccidians (Coccidia, Apicomplexa) and haemosporidians (Haemosporida, Apicomplexa). However, our data do not clarify whether these organisms are opportunistically present in the ulcerations or potentially involved in the development of the disease.

The results of microscopy analyses (i.e. light and electron) allow us to better understand the ulcer appearance but, also, the tissue disturbance within these ulcers. First, the only work analyzing the ultrastructure of the integument of a sea cucumber—of the *Holothuria* genus—is that of VandenSpiegel et al*.*^35^ who were interested in the dorsal papillae of the temperate species *Holothuria forskali*. The papilla epidermis of *H. forskali* is a monostratified epithelium covered by a well-marked cuticle and is made up of four cell types that are pigment cells, mucocytes and two types of ciliated cells. The first two cell types are found in the general epidermis of the *H. scabra* dorsal integument with a third cell type, the covering cells. The epidermis of *H. scabra* dorsal integument is extremely thin, of the order of a few hundred nanometres over a large part of the body surface. It is therefore likely that a stressed animal, mechanically or physiologically, would be easily affected by alteration of this thin epidermal coating leading to the exposure of the dermis to the surrounding environment. The ulcerated individuals react by forming a plug of collagen fibres from the dense connective tissue, and in some cases, opportunistic bacteria can settle in the dermis and, probably, cause the extent of the lesion to progress.

From the data we have collected, a likely cause of the SKUD *Hs-MG-20* is the repeated and prolonged exposures to cold temperatures. Over two years of monthly follow-up, the disease prevailed twice, most of the time from May to September with, for the first year, a peak in July. The period from May to September is the coolest period when the water does not exceed 26 °C on average. Besides, at that time, the average minimum air temperature recorded during these months did not reach 16 °C. Furthermore, the exposure of the sea pens is important when approaching the spring tide, the height of the water above the substrate reaching no more than 20 cm. It is therefore very likely that sea cucumbers will be subjected to very low temperatures several times a month. In this context, Kühnhold et al*.* recently showed that a good range of temperature is essential during the rearing of *H. scabra* and that they cannot survive temperatures below 22 °C or above 38°C^43,44^. Between 22 and 38 °C, *H*. *scabra* has been shown to have positive aerobic capacity and its peak performance is potentially between 29 and 31.5 °C^43^. Between 39 and 41 °C, the sea cucumber decreased its respiration. In parallel, Hsp 70 gene expression levels significantly increase at 41 °C indicating the presence of a heat shock response and homeostatic disruption. Between 17 and 22 °C, homeostatic disruption is also observed and potentially associated to an increase of the energetic expenses ensuring the basal maintenance costs^43^. Additional experiments should be performed to evaluate the direct impact of temperature changes (in particular, repeated or prolonged exposures to cold temperatures) on the health of *H. scabra* sea cucumbers in an aquaculture context. In the present work, it should be noted that enclosures H and I, those located closest to the coast, are the most impacted by the SKUD. Sea cucumbers subject to these conditions could see their fitness reduced, resulting in a less effective natural defense against the invasion of opportunistic microorganisms including bacteria, fungi or viruses, which at the end would result in the development of this disease. Additional work would be necessary to better characterise the environmental conditions of the different enclosures and to interpret potential differences in the emergence of SKUD events within the different enclosures.

An abiotic cause of the SKUD *Hs-MG-20* is likely. Functional gene expression analyses would be of interest to better understand the changes occurring within the sea cucumber integument during the *Hs-MG* disease. Transcriptomic analyses performed on *A. japonicus* recently informed on functional gene expression^45^ but also on microRNAs expression^46,47^. To our knowledge, such analyses have not been performed on *H. scabra,* yet.

## Supplementary Information

Supplementary Information.

Supplementary Table S1.

Supplementary Table S2.

## Data Availability

Metagenomic sequencing data (i.e. raw reads) used in this study were submitted on the NCBI database under the BioProject PRJNA634538 and the following Biosample names: SAMN14999041, SAMN14999042, SAMN14999043, SAMN14999044. Metagenome sequence samples also exist as public records that were assigned the following IDs in MG-RAST databases: mgm4871537.3, mgm4871625.3, mgm4880040.3, mgm4883751.3 (MG-RAST project: http://www.mg-rast.org/linkin.cgi?project=mgp90921). Sequences and strain identifications of the three cultures were submitted on GenBank: culture 1, *Vibrio* sp. SKUD-MD-20 C1, GenBank: MT416091; culture 2, *Vibrio variabilis* SKUD-MD-20 C2*,* GenBank: MT416092; culture 3, *Vibrio parahaemolyticus* SKUD-MD-20 C3, GenBank: MT416093.
