# Supplementary material for: Epidemiology of a SKin Ulceration Disease (SKUD) in the sea cucumber Holothuria scabra with a review on the SKUDs in Holothuroidea (Echinodermata)
Source: Sci Rep. 2020 Dec 17;10:22150. doi: 10.1038/s41598-020-78876-0 (PMC7746772; doi:10.1038/s41598-020-78876-0)
Supplement: Supplementary file 1 — Supplementary Information. [file 41598_2020_78876_MOESM1_ESM.pdf]

<u>Title:</u> Epidemiology of a SKin Ulceration Disease (SKUD) in the sea cucumber *Holothuria scabra* with a review on the SKUDs in Holothuroidea (Echinodermata)

Jérôme Delroisse[1,2], Kévin Van Wayneberghe[1], Patrick Flammang[1], David Gillan[3], Pascal Gerbaux[4], Noel Opina[5], Gildas Georges Boleslas Todinanahary[2,5], Igor Eeckhaut[1,2,5].

[1] Biology of Marine Organisms and Biomimetics, Research Institute for Biosciences, University of Mons – UMONS, Place du Parc, 6, 7000, Mons, Belgium.
[2] Marine Station of Belaza, Institut Halieutique et des Sciences Marines (IH.SM), University of Toliara, Route du Port Mahavatse II, P.O. Box 141, 601 Toliara, Madagascar
[3] Proteomics and Microbiology Lab, Research Institute for Biosciences, University of Mons – UMONS, Place du Parc, 6, 7000, Mons, Belgium.
[4] Organic Synthesis and Mass Spectrometry Lab, Interdisciplinary Center for Mass Spectrometry, Research Institute for Biosciences, University of Mons – UMONS, Place du Parc, 6, 7000, Mons, Belgium.
[5] Madagascar Holothurie (R&D of Indian Ocean Trepang), Toliara, Route du Port Mahavatse II, P.O. Box 141, 601 Toliara, Madagascar

Correspondence and requests for materials should be addressed to J.D. (email: Jerome.Delroisse@umons.ac.be) and I.E. (email: Igor.Eeckhaut@umons.ac.be)

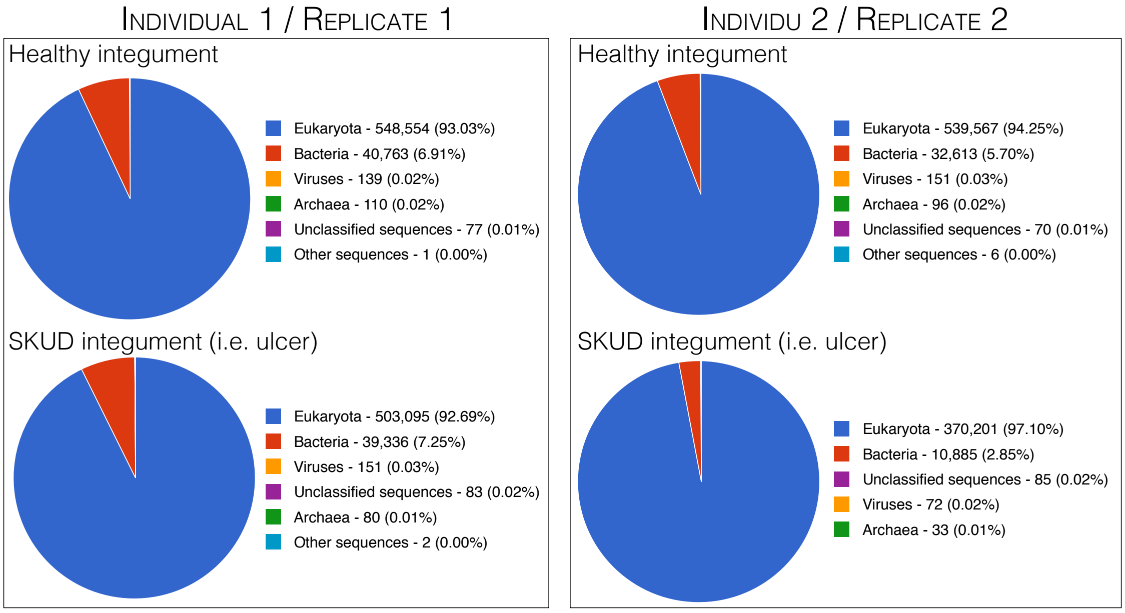

Supplementary Figure S1: Proportions of life domains based on RefSeq annotation (MG-rast parameters: e-value threshold: >e^{-10}, identity threshold: >70%, alignment length > 15bp, read abundancy > 1, best hits are shown).

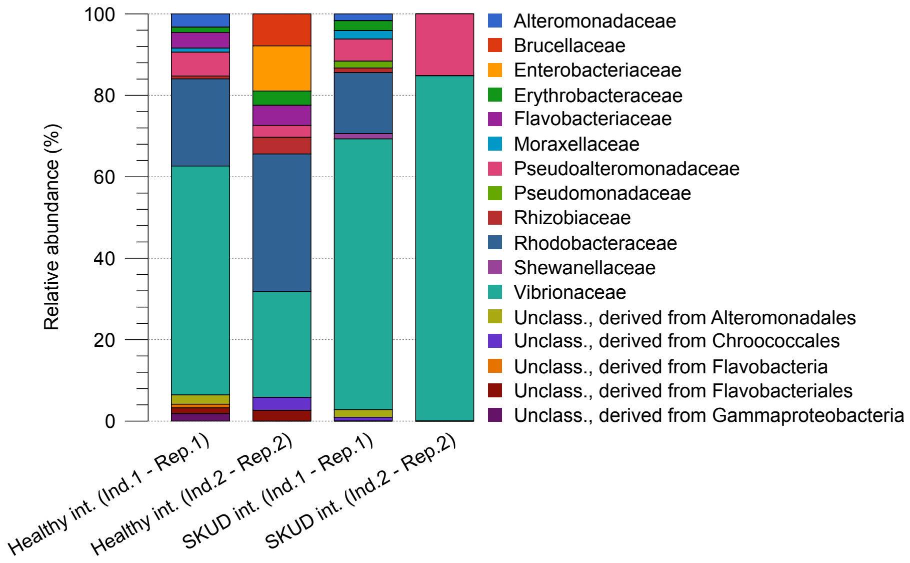

Supplementary Figure S2: Proportions of eubacterial families based on RefSeq annotation (MG-rast parameters: e-value threshold: >e^{-40}, identity threshold: >85%, alignment length > 15bp, read abundancy > 5, best hits are shown).

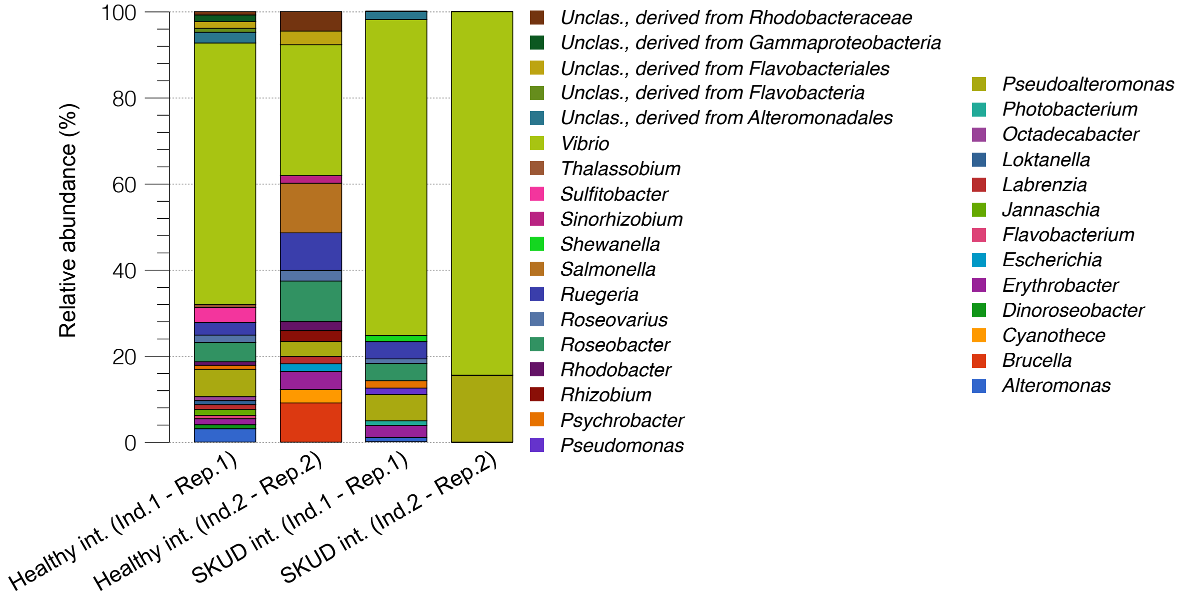

Supplementary Figure S3: Proportions of eubacterial genera based on RefSeq annotation (MG-rast parameters: e-value threshold: >e^{-40}, identity threshold: >85%, alignment length > 15bp, read abundancy > 5, best hits are shown).

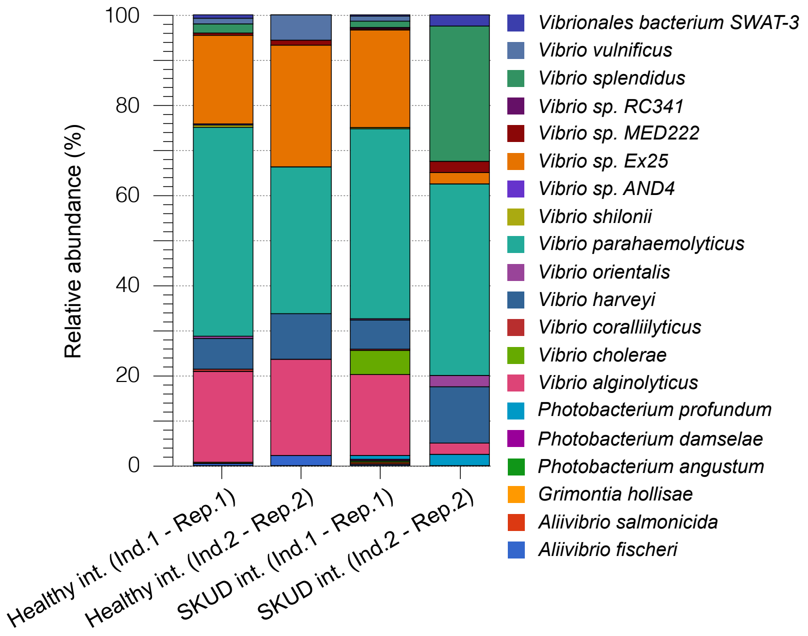

Supplementary Figure S4: Proportions of vibrionale species based on RefSeq annotation (MG-rast parameters: e-value threshold: >e^{-40}, identity threshold: >85%, alignment length > 15bp, read abundancy > 1, best hits are shown).

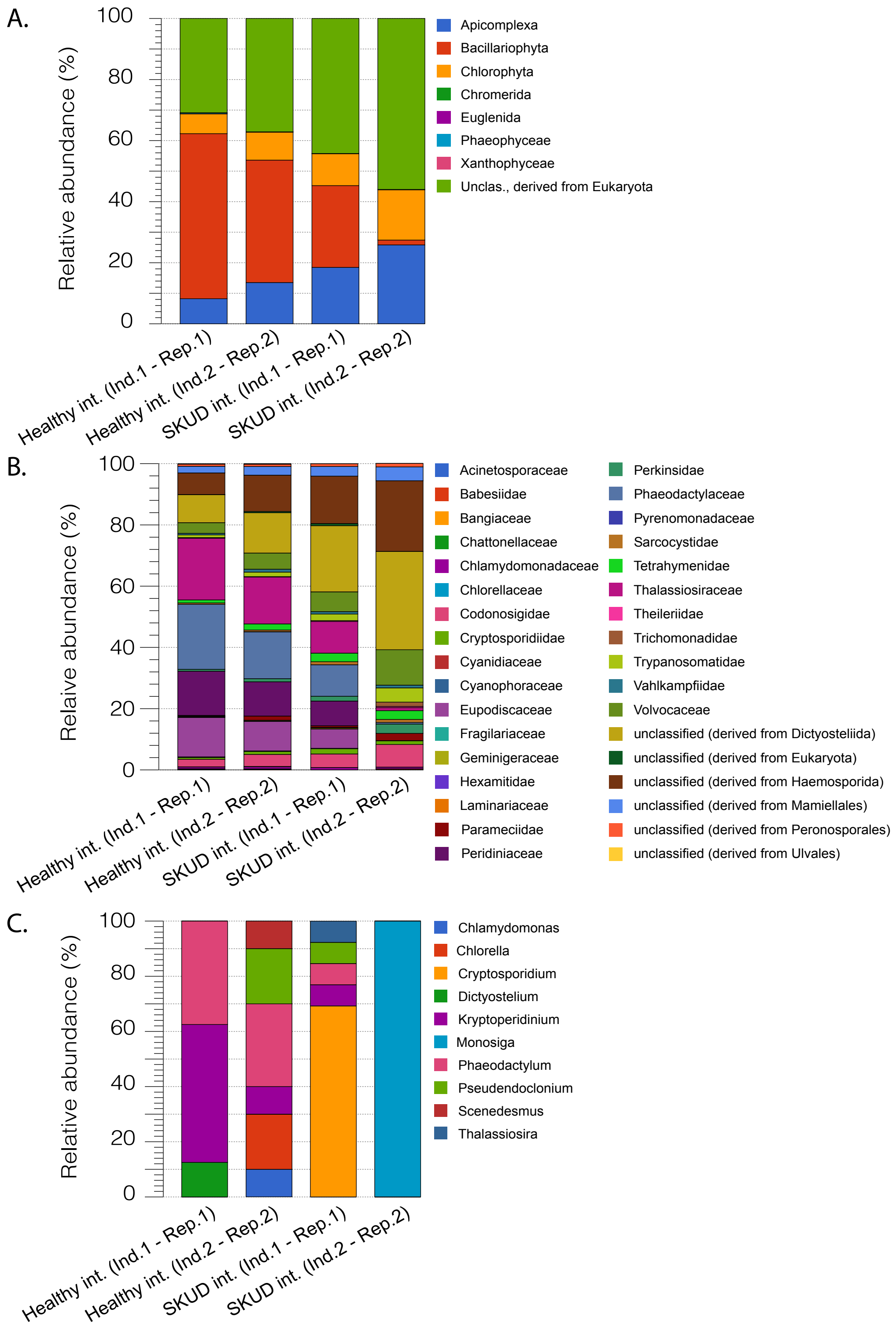

Supplementary Figure S5: Proportions of Eukaryota (excluding Metazoa, Fungi and Streptophyta) phyla (A), families (B) and genera (C) based on RefSeq annotation (MG-rast parameters for phyla and families proportions: e-value threshold: >e^{-10}, identity threshold: >70%, alignment length > 15bp, read abundancy > 1, best hits are shown; MG-rast parameters for genera proportion: e-value threshold: >e^{-40}, identity threshold: >85%, alignment length > 15bp, read abundancy > 1, best hits are shown).

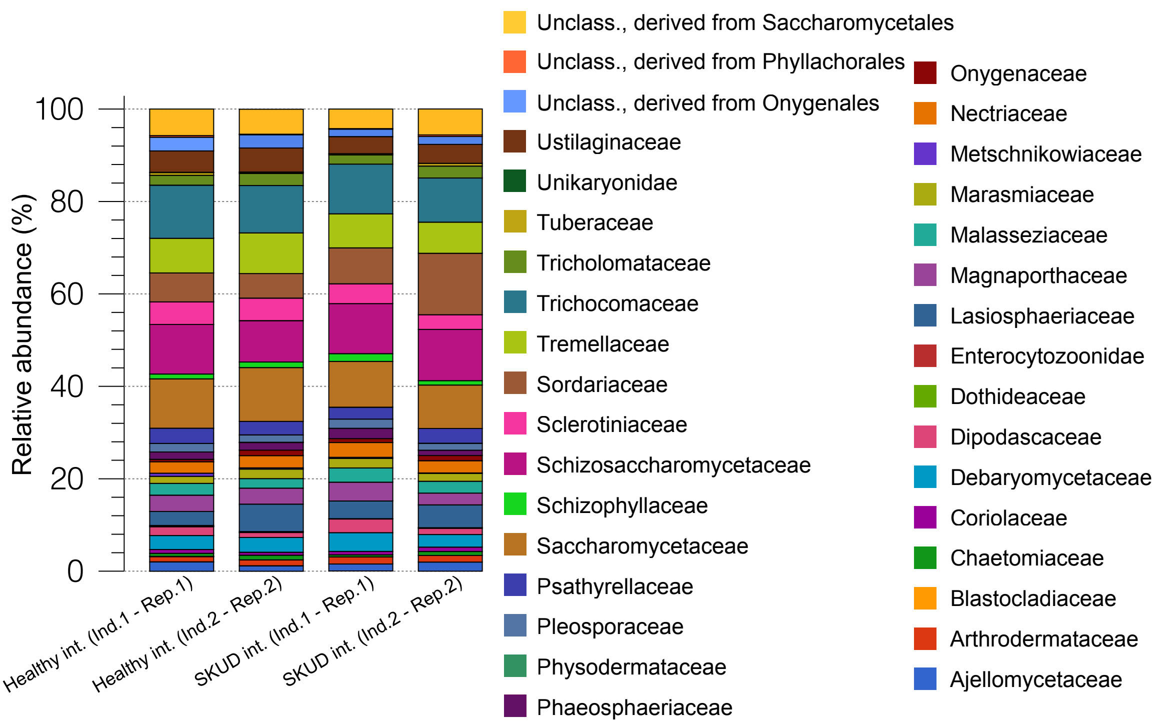

Supplementary Figure S6: Proportions of Fungi families based on RefSeq annotation (MG-rast parameters: e-value threshold: >e^{-10}, identity threshold: >70%, alignment length > 15bp, read abundancy > 1, best hits are shown).

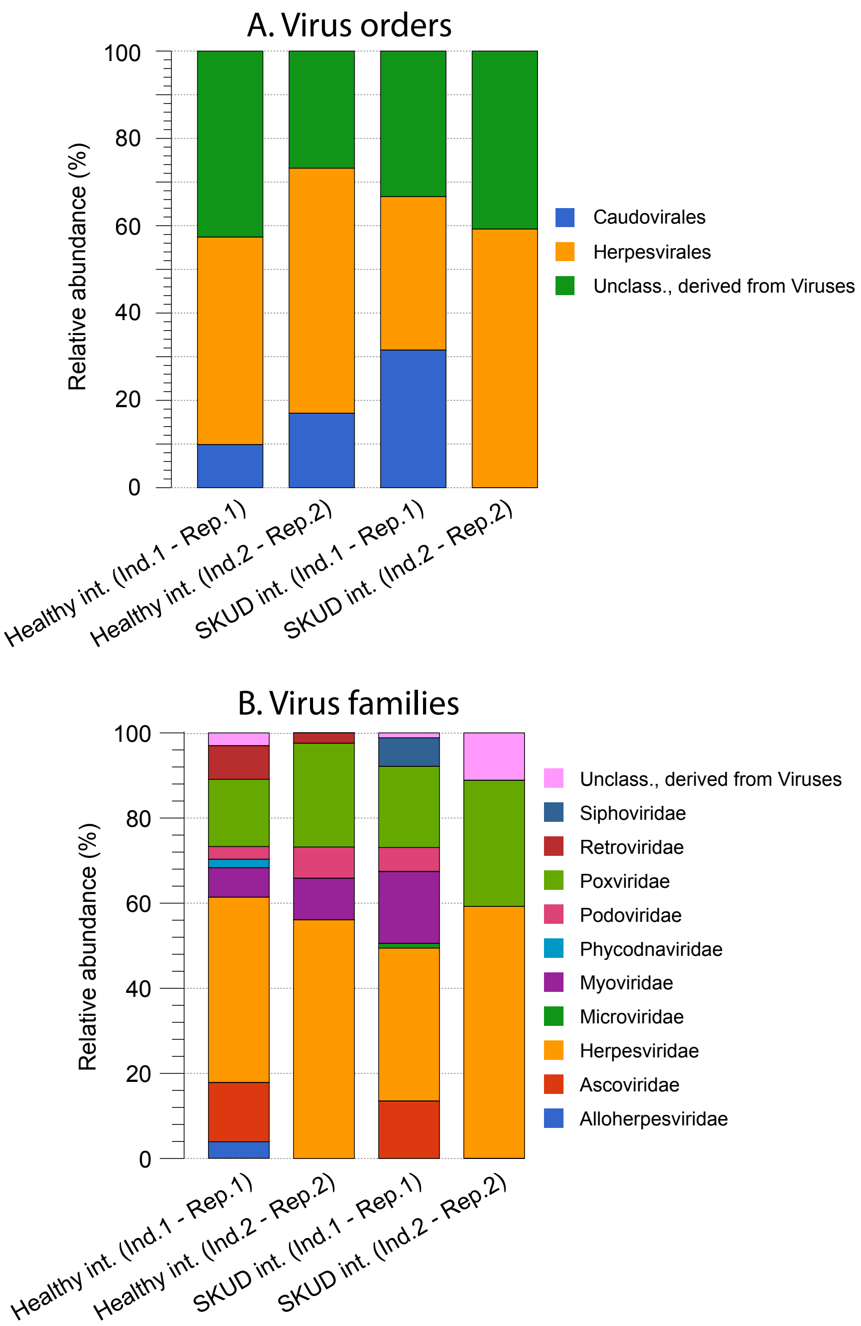

Supplementary Figure S7: Proportions of Virus orders and families based on RefSeq annotation (MG-rast parameters: e-value threshold: >e^{-10}, identity threshold: >70%, alignment length > 15bp, read abundancy > 1, best hits are shown).

BLASTn query	Description	Accession
Query	*Vibrio* sp. SKUD-MD-20 C1 (culture 1)	MT416091

BLASTn output	Description	Max Score	Total Score	Query Cover	E value	Per. Ident	Accession
1	*Vibrio alginolyticus strain YWT7-80 16S ribosomal RNA gene, partial sequence*	625	625	100%	5,00E-175	99.42%	MT368033.1
2	*Vibrio alginolyticus strain YWO6-72 16S ribosomal RNA gene, partial sequence*	625	625	100%	5,00E-175	99.42%	MT368030.1
3	*Vibrio alginolyticus strain YWT6-68 16S ribosomal RNA gene, partial sequence*	625	625	100%	5,00E-175	99.42%	MT368029.1
4	*Vibrio alginolyticus strain YWO3-29 16S ribosomal RNA gene, partial sequence*	625	625	100%	5,00E-175	99.42%	MT368021.1
5	*Vibrio alginolyticus strain YWO3-27 16S ribosomal RNA gene, partial sequence*	625	625	100%	5,00E-175	99.42%	MT368020.1
6	*Vibrio alginolyticus strain YPL2-20 16S ribosomal RNA gene, partial sequence*	625	625	100%	5,00E-175	99.42%	MT368017.1
7	*Vibrio sp. strain BC76 16S ribosomal RNA gene, partial sequence*	625	625	100%	5,00E-175	99.42%	MT325889.1
8	*Vibrio neocaledonicus strain BC43 16S ribosomal RNA gene, partial sequence*	625	625	100%	5,00E-175	99.42%	MT325882.1
9	*Vibrio neocaledonicus strain BC41 16S ribosomal RNA gene, partial sequence*	625	625	100%	5,00E-175	99.42%	MT325880.1
10	*Vibrio sp. strain BC37 16S ribosomal RNA gene, partial sequence*	625	625	100%	5,00E-175	99.42%	MT325879.1

BLASTn query	Description	Accession
Query	*Vibrio variabilis* SKUD-MD-20 C2	MT416092

BLASTn output	Description	Max Score	Total Score	Query Cover	E value	Per. Ident	Accession
1	*Vibrio variabilis strain T01 16S ribosomal RNA gene, partial sequence*	619	619	100%	2,00E-173	99.13%	KP329555.1
2	*Uncultured bacterium clone GXTJ5A301A0EAG 16S ribosomal RNA gene, partial sequence*	619	619	100%	2,00E-173	99.13%	KF331146.1
3	*Uncultured bacterium clone GXTJ5A301BZLBJ 16S ribosomal RNA gene, partial sequence*	619	619	100%	2,00E-173	99.13%	KF330715.1
4	*Uncultured bacterium clone GXTJ5A301A9XNS 16S ribosomal RNA gene, partial sequence*	619	619	100%	2,00E-173	99.13%	KF330671.1
5	*Bacterium 3H301 16S ribosomal RNA gene, partial sequence*	616	616	100%	3,00E-172	98.84%	JF411542.1
6	*Vibrio sp. strain MW-23 16S ribosomal RNA gene, partial sequence*	614	614	100%	1,00E-171	98.84%	MG686115.1
7	*Uncultured bacterium clone GXTJ5A301BX87K 16S ribosomal RNA gene, partial sequence*	614	614	100%	1,00E-171	98.84%	KF332760.1
8	*Vibrio sinaloensis strain F5C1 16S ribosomal RNA gene, partial sequence*	614	614	100%	1,00E-171	98.84%	JX999951.1
9	*Uncultured bacterium clone GXTJ5A301AMHXZ 16S ribosomal RNA gene, partial sequence*	612	612	100%	4,00E-171	98.84%	KF333857.1
10	*Uncultured bacterium clone GXTJ5A301BXNPN 16S ribosomal RNA gene, partial sequence*	612	612	100%	4,00E-171	98.84%	KF333347.1

BLASTn query	Description	Accession
Query	*Vibrio parahaemolyticus* SKUD-MD-20 C3	MT416093

BLASTn output	Description	Max Score	Total Score	Query Cover	E value	Per. Ident	Accession
1	*Vibrio parahaemolyticus strain TY-49 16S ribosomal RNA gene, partial sequence*	608	608	100%	5,00E-170	98.55%	MN960078.1
2	*Vibrio alginolyticus strain JK158 16S ribosomal RNA gene, partial sequence*	608	608	100%	5,00E-170	98.55%	MK696466.1
3	*Vibrio alginolyticus strain JK140 16S ribosomal RNA gene, partial sequence*	608	608	100%	5,00E-170	98.55%	MK696461.1
4	*Vibrio sp. strain 201707CJKOP-Y194 16S ribosomal RNA gene, partial sequence*	608	608	100%	5,00E-170	98.55%	MG593758.1
5	*Vibrio sp. strain 201707CJKOP-25 16S ribosomal RNA gene, partial sequence*	608	608	100%	5,00E-170	98.55%	MG309461.1
6	*Vibrio sp. strain 24/486Kan 16S ribosomal RNA gene, partial sequence*	608	608	100%	5,00E-170	98.55%	MK512027.1
7	*Vibrio alginolyticus strain FDAARGOS_108 chromosome 1, complete sequence*	608	7054	100%	5,00E-170	98.55%	CP014053.1
8	*Vibrio sp. strain pSSB-01 16S ribosomal RNA gene, partial sequence*	608	608	100%	5,00E-170	98.55%	MH315831.1
9	*Vibrio parahaemolyticus strain Vp-D9 16S ribosomal RNA gene, partial sequence*	608	608	100%	5,00E-170	98.55%	MH298547.1
10	*Vibrio parahaemolyticus strain msr11 16S ribosomal RNA gene, partial sequence*	608	608	100%	5,00E-170	98.55%	MH244245.1

Supplementary Table S3: BLAST analysis results of 16S ribosomal RNA gene obtained from isolated cultured bacteria.
